# The Association of Oral Processing Factors and Nutrient Intake in Community-Dwelling Older Adults: A Systematic Review and Meta-Analysis

**DOI:** 10.1093/nutrit/nuae080

**Published:** 2024-06-25

**Authors:** Supatchayaporn Nitsuwat, James Webster, Anwesha Sarkar, Janet Cade

**Affiliations:** Nutritional Epidemiology Group, School of Food Science and Nutrition, Faculty of Environment, University of Leeds, Leeds, LS2 9JT, United Kingdom; Nutritional Epidemiology Group, School of Food Science and Nutrition, Faculty of Environment, University of Leeds, Leeds, LS2 9JT, United Kingdom; Food Colloids and Bioprocessing Group, School of Food Science and Nutrition, Faculty of Environment, University of Leeds, Leeds, LS2 9JT, United Kingdom; Nutritional Epidemiology Group, School of Food Science and Nutrition, Faculty of Environment, University of Leeds, Leeds, LS2 9JT, United Kingdom

**Keywords:** aging population, denture status, mastication, nutrition, dietary intake

## Abstract

**Context:**

Oral health and food oral-processing issues emerge with functional decline in the older adult population, potentially increasing the risk of malnutrition. Impairment of oral health is associated with poorer nutrition status; however, the relationship between oral factors and the intake of each nutrient remains poorly understood.

**Objective:**

The associations between different oral factors and nutrient intakes among community-dwelling older adults were investigated.

**Data Sources:**

A literature search from 5 databases (Web of Science, Scopus, Cochrane Library, Ovid [MEDLINE and Embase], and CINAHL) was completed on February 1, 2022. The search was limited to peer-reviewed articles published between the years 2012 and 2022.

**Data Extraction:**

Six cross-sectional studies were included in the meta-analysis. Two authors independently completed the data extraction and summarized the study characteristics, factors adjusted for in the statistical analysis, the outcome, and summary statistics of the results.

**Data Analysis:**

Meta-analyses showed evidence of a significant association between compromised oral factors (namely, denture status, chewing ability, and the number of teeth) with lower energy (weighted mean difference [WMD], –107 kcal d^–1^ (95% CI, –132 to –81), protein (WMD, –5.2 g d^–1^; 95% CI, –6.6 to –3.8), fat (WMD, –4.6 g d^–1^; 95% CI, –6.7 to –2.6), carbohydrate (WMD, –8.8 g d^–1^; 95% CI, –13.9 to –3.7), and vitamin C intakes (WMD, –12.9 mg d^–1^; 95% CI, –16.6 to –9.2) in older adults.

**Conclusion:**

Oral health can be an indicator of compromised daily energy, protein, fat, carbohydrate, and vitamin C intakes in older adults. However, the small sample size of the studies included in this review and the heterogeneity among macronutrient studies should be considered. Because of the lack of studies covering all aspects of food oral processing (eg, salivary flow rate, tongue pressure), the associations between oral processing and nutrient intake were not thoroughly explored.

**Systematic Review Registration:**

PROSPERO registration no. CRD42022308823.

## INTRODUCTION

The global population is aging; the increase in the population median age is attributed to declining birthrates and increasing life expectancy.^1^ Although individuals are living longer, data indicate that the proportion of years lived in a state of disability or illness is also increasing.^2^ Noncommunicable diseases are identified as the leading cause of premature death and disability, and preventive measures include healthy behaviors in terms of physical activity and nutrition, with evidence suggesting the possibility of reducing the risk of these events.^3^ Although energy intake may decrease with age, the nutritional needs of older adults do not differ much from those of younger people.^3^ However, older adults tend to lack awareness regarding their nutritional needs and the consequences of not meeting them. A high risk of malnutrition was found in 8.5% of European community-dwelling older adults and as high as 45.5% in those institutionalized in hospitals and care homes combined.^4^^,^^5^ According to results of a survey by the British Association of Parenteral and Enteral Nutrition, malnutrition was more prevalent in adults aged 65 years or older (32%) than in those younger than 65 years (24%).^6^ However, the numbers could be higher because malnutrition that occurs mostly in older peoples’ homes remains undetected.^7^ Not only will malnutrition impact the long-term health conditions of older adults, but it can also burden the healthcare system through hospital admissions and prolonged stays.^8^ Therefore, prevention is crucial.

Aging results in the decline of several physiological functions that can impair nutritional status. The decline of motor functions, orofacial muscular capabilities, sensory functions, eating capability, and limited ability to secrete gastrointestinal enzymes and bile acids can negatively affect appetite and absorption of nutrients, thus affecting the nutritional status.^9^ Often, oral health and eating capability decline cause discomfort and difficulties in chewing, salivation, and swallowing, as well as limited ability to perceive certain tastes and textures, affecting nutritional intake.^10^ Among various eating capabilities, denture status, which is linked to bite force, is the most-studied factor in relation to nutrient intake.^11^

Several studies of older adults have suggested that wearing dentures and having poor dental health can decrease nutrient intake, thereby increasing the risk of malnutrition, compared with those without compromised dental status.^12–15^ However, the levels of nutrient intake negatively affected by compromised dental status were reported inconsistently between studies. Decreased intakes of nutrients were reported in denture wearers compared with those who were dentate without dentures.^12^ Despite this, another study found no significant difference in nutrient intake among those who had some natural teeth, irrespective of their denture status.^15^ Interestingly, a study that investigated the effects of denture status and self-perceived chewing ability on nutrient intakes found that rather than dentures, it was chewing ability that was associated with lower intakes of vegetables and fruits.^16^ Another study further illustrated that self-perceived chewing problems and oral discomfort can lead to avoidance of foods and digestive complaints more so than objectively measured dental status.^17^ Therefore, the overall oral processing (ie, the initial process of breakdown of foods that includes stages of first bite, mastication, mixing with saliva, bolus formation, and swallowing^18^) is an important aspect that may affect nutrient intake, but, to our knowledge, has not been studied systematically. In this systematic literature review and meta-analysis, we comprehensively explored existing literature and investigated the association of oral processing factors, such as denture status, chewing ability, occlusal force, and the number of teeth, with the intakes of energy, macronutrients (namely, carbohydrate, fat, and protein), and micronutrients (namely, iron, vitamin C, calcium, and potassium) by older adults.

## MATERIALS AND METHODS

This study was registered in the International Prospective Register of Systematic Reviews (PROSPERO; registration no. CRD42022308823). The systematic literature search was conducted following the Preferred Reporting Items for Systematic Review and Meta-Analyses (PRISMA) guidelines (the PRISMA checklist is provided in Table S1).^19^

### Search strategy

The search was conducted using 5 databases: Web of Science, Scopus, Cochrane Library, Ovid (MEDLINE and Embase), and CINAHL; and limited to peer-reviewed articles written in English and published in the past 10 years (2012–2022). The search strategy was defined following the research question formulated according to the PICOS (Population, Intervention, Comparison, Outcomes, Study design) framework, as shown in Table 1.

**Table 1.** PICOS Criteria for Inclusion of Studies

Parameters	Inclusion criterion
Population	Community-dwelling older adults aged ≥65 y who are cognitively healthy and without diagnosed xerostomia
Intervention	Good or optimal oral processing conditions, both objectively and subjectively measured
Comparison	Poor or below-optimal oral processing conditions, both objectively and subjectively measured
Outcome	Energy and nutrient intakes
Study design	All types of study designs except meta-analyses, systematic reviews, ideas, editorials, opinions, animal research studies, and studies that focus on certain medical conditions of the population

Search terms included older adults, elderly, aged care, aging, oral processing, chew/chewing, mastication, bite/biting force, bite/biting strength, tongue pressure, tongue force, denture, dentition, saliva, salivation, bolus, saliva production, salivary flow, food intake, nutrient intake, diet, dietary, energy intake, and food consumption. The terms were searched for in the title and abstracts (the full search query for each database is provided in Table S2). The search was completed by 1 author (S.N.) on February 1, 2022.

### Screening and inclusion of studies

The title and abstract of the search results were downloaded and managed using the EndNote 20 software (Clarivate, Philadelphia, PA).^20^ Duplicates were filtered out using the software and cross-checked to manually remove any that were missed.

The remaining records were screened using the following criteria: (1) study population was noninstitutionalized, cognitively healthy human older adults without diagnosed xerostomia, aged 65 years old or older; (2) inclusive of all study designs and excluding meta-analyses, systematic reviews, ideas, editorials, opinions, and studies that focused on a particular medical condition; (3) categorized the study population on the basis of any oral processing–related factors both subjectively or objectively (eg, mastication performance, chewing ability, bite force, tongue pressure, salivary flow rate), and/or denture status (ie, complete denture, partial denture, and implants); and (4) measured energy and nutrient intake as outcomes.

Screening of the titles and abstracts was independently conducted in duplicate by 3 authors (S.N., J.C., and A.S.). All titles and abstracts were screened by 1 author (S.N.), and the second screening was shared between 2 (J.C. and A.S.). Cohen’s κ was used to assess the reliability between the reviewers.^21^ The full-text screening was conducted by 1 author (S.N.), and the results and reasons for exclusion were discussed with 2 other authors (J.C. and A.S.) to reach a consensus on inclusion or exclusion.

### Data extraction and quality assessment

The data from the studies that met the inclusion criteria were extracted independently by 2 authors (S.N. and J.W.). Microsoft Excel (Redmond, WA) spreadsheets were used to collect data about the study author(s), study publication year, characteristics of the participants (ie, total number of participants, setting, age, and sex), the study design, whether it was a national sample or not, how the participants were categorized, the factors that were adjusted for in the statistical analysis, the outcome, and the summary statistics of the results. Because all the studies were cross-sectional, study quality was independently assessed using the Joanna Briggs Institute Critical Appraisal tool checklist for analytical cross-sectional studies.^22^ Two authors (S.N. and J.W.) independently assessed the studies by answering 8 questions from the checklist, with disagreements resolved by consensus. Answers could be given as yes, no, unclear, or not applicable, and the study quality was determined by the percentage of yes answers, with 49% or less considered low, 50% to 69% as moderate, and 70% and above as high.^23^

### Statistical analysis

Meta-analyses were performed using the Review Manager (RevMan) 5.4 software (Cochrane, London, UK)^24^ with studies pooled based on the outcomes. The list of nutrients reported was not standardized across studies; therefore, meta-analysis was performed only if the outcome was included in 2 or more studies. The unit used for analysis was kilocalories per day for energy intake, and those reported as kilojoules were converted to kcal by dividing with the conversion factor 4.184.^25^ Macronutrients were analyzed as grams per day and micronutrients as milligrams per day, with those reported otherwise converted to these units. Arithmetic means and SDs were the main reported format; other formats were converted before data pooling. Geometric means and CIs were converted to arithmetic means using the method of Higgins et al,^26^ and SEs were converted to SDs by multiplying with the square root of the sample size.^27^ The overall effect and its 95% CI were determined by the weighted mean differences (WMDs) between the control and comparison groups in each study and their SD. The *I*^2^ statistic was used to determine the heterogeneity of the studies, with values of 50% or greater treated as having substantial heterogeneity, using a random-effects model. A fixed-effects model was applied to those with lower *I*^2^ values.

Subgroup analyses were conducted based on the type of oral factor, even with only 2 studies included, given the lack of studies in the area of interest. According to the Cochrane Consumers and Communication Group reviews,^28^ 2 studies can sufficiently support a meta-analysis if their findings are notably similar and can be effectively combined. In addition, we considered the sample size and study design of each study, and caution was exercised in interpreting and reporting the results, with heterogeneity accounted for. If a study contained more than 1 experimental group, the groups were combined, where appropriate, to create a single pair-wise comparison as recommended by the Cochrane guidelines.^29^ Because living alone is associated with an increased risk of nutrient deficiencies and depression,^30^^,^^31^ a sensitivity analysis was undertaken by excluding the study of Kim and Jin,^30^ which included participants known to live with at least 1 other family member.

## RESULTS

### Study selection

**Figure 1.**
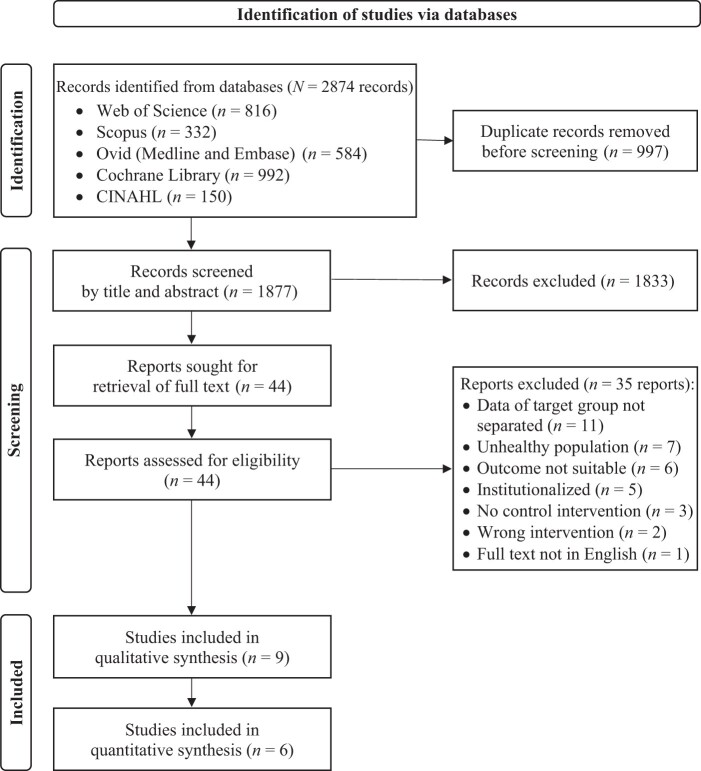
Preferred Reporting Items for Systematic Review and Meta-Analyses Flow Diagram of the Study Selection Process

A total of 2874 records were recovered from the databases, as detailed in the PRISMA flow diagram (Figure 1). After the removal of 997 duplicates, the titles, and abstracts of the remaining 1877 records were screened, which resulted in 44 records that were sought for the full text, with moderate (Cohen’s κ = 0.56) to substantial agreement (Cohen’s κ = 0.77) between reviewers. The full texts of all 44 records were retrieved and screened against the criteria. Another 35 reports then were excluded with reason (as detailed in Table S3), leaving a total of 9 studies for data extraction and quality assessment.

### Study characteristics and quality assessment

The characteristics of studies that passed the screening criteria are detailed in Table 2,^15^^,^^30^^,^^32–38^, and additional metrics of the results presented in the included studies are provided in Table S4. All were cross-sectional studies published between the years 2014 and 2021. Studies were mostly national samples (*n* = 6) from South Korea (*n* = 4), Japan (*n* = 2), Australia (*n* = 1), Israel (*n* = 1), and the United Kingdom (*n* = 1). The sample size ranged from 509 to 3751 participants, including both men and women; 1 study was conducted only with male participants,^36^ and another did not specify participants’ sex.^38^ Exposures assessed were denture status (*n* = 4), chewing ability (*n* = 3), number of teeth (*n* = 2), occlusal force (*n* = 1), and the posterior functional teeth units (*n* = 1).

**Table 2.** Characteristics of Studies Included in the Review

Reference	Participants					Study design	National sample	Exposure and method of ascertainment	Outcome measured
	*N*	Setting	Age, y	Sex. no.					
				Male	Female				
Choi et al (2014)^32^	3751	South Korea	≥65	302	420	Cross-sectional	KNHANES, 2007	Prosthodontic status No dentures or with fixed prosthesis With partial or full dentures Clinical examination by trained dentist	Dietary intake by 24-h recall
Han and Kim (2016)^33^	1168	South Korea	Mean ± SD: 75.11 ± 6.1	454	714	Cross-sectional	KNHANES 2008–2010	1. Denture status 1.1. With dentures 1.2. Without dentures 2. Edentulism 2.1. One edentulous arch 2.1.1. With dentures 2.1.2. Without dentures 2.2. Both edentulous arch 2.2.1. With dentures 2.2.2. Without dentures Clinical examination by trained dentist	Dietary intake by 24-h recall
Inomata et al (2017)^34^	760	Japan	79–81	364	396	Cross-sectional	Not a national sample	1. Number of teeth 1.1. low (0–9 teeth) 1.2. mid (10–22 teeth) 1.3. high (≥23 teeth) 2. Occlusal force 2.1. low (0–9 teeth) 2.2. mid (10–22 teeth) 2.3. high (≥23 teeth) Clinical examination by trained dentist	Brief self-administered diet history questionnaire
Kim and Jin (2018)^30^	2904	South Korea	65–84	1250	1654	Cross-sectional	KNHANES, 2013–2015	Number of natural teeth by living status 1. Living alone 1.1. 0–10 teeth 1.2. 11–20 teeth 1.3. Over 21 teeth 2. Living with family 2.1. 0–10 teeth 2.2. 11–20 teeth 2.3. >21 teeth Clinical examination by trained dentist	Dietary intake by 24-h recall
Kwon et al (2017)^35^	999	South Korea	≥65	423	576	Cross-sectional	KNHANES 2013	Chewing ability Normal (reported as very easy to moderate) With difficulty(reported as difficult to very difficult) Self-reported questionnaire	Dietary intake by 24-h recall
Milledge et al (2021)^36^	608	Australia	Mean ± SD: 83.9 ± 4.1	608	0	Cross-sectional	Not national samples	Posterior FTUs Only natural FTUs Mixture of natural and replaced FTUs Only replaced FTUs Clinical examination of opposing molars and premolars by trained oral health therapists	Open-ended, structured questionnaire adapted from Sydney Southwest Area Health Service outpatient diet history form
Motokawa et al (2021)^37^	509	Japan	Mean age ± SD by chewing ability: Poor = 73.9 ± 5.8 y Good = 72.1 ± 5.1 y	215	294	Cross-sectional	Not national samples	Chewing ability Poor chewing ability (color-change level 1–3) Good chewing ability (color-change level 4–5) Color-changeable chewing gum test	Dietary intake by semi-quantitative food frequency questionnaire
Natapov et al (2018)^38^	1776	Israel	≥65	Not specified	Not specified	Cross-sectional	National Health and Nutrition Survey of the Elderly (Mabat Zahav, 2005–2006)	1. Dentures: none, partial, or full 2. Chewing problems: yes, no Self-reported questionnaire	Dietary intake by 24-h recall
Watson et al (2019)^15^	1053	United Kingdom	Mean ± SD: 74.1 ± 6.9	432	621	Cross-sectional	UK National Diet and Nutrition Survey (2008–2014)	Denture status: Edentate with dentures Dentate with dentures Dentate without dentures Computer assisted personal interview	Estimated 4-d food diary, corroborated by blood biomarkers

*Abbreviations:* FTU, functional tooth unit; KNHANES, Korean National Health and Nutrition Examination Survey.

The different oral-factor participant groups compared in the included studies are summarized in Table 3. The World Health Organization Global goals for oral health in 2020 defined functional dentition as having at least 21 teeth.^39^ Therefore, for this meta-analysis, we used 21 natural teeth as the cutoff value to categorize participants into control and comparison groups based on the number of teeth. To obtain the dietary intake data from participants, the 24-hour dietary recall (*n* = 5) was the method most applied. Other methods included the diet history questionnaire (*n* = 2), semi-quantitative food frequency questionnaire (*n* = 1), and the 4-day food diary (*n* = 1).

**Table 3.** Summary of Oral Factor Controls and Comparison from the Included Studies

Oral factors	Control	Comparison
Denture status	No dentures	With dentures (dentate and edentate)
Edentulism status	Edentulous, with denture	Edentulous, without denture
Chewing ability	Normal or good chewing ability	With difficulty or poor chewing ability
No. of teeth	Having ≥21 teeth	Having <21 teeth (0–20 teeth)
Molars and premolars status	Natural opposing molars and premolars	Artificial or mixed opposing molars and premolars

The quality of all studies in this systematic review ranged from moderate to high (50%–88%), as detailed in the Table S5. The quality of 3 studies was rated as moderate^35^^,^^37^^,^^38^ and that of the remaining 6 was high. The strength of these studies lay in the detailed description of the participants as well as the identification of confounders and the strategies used to address them. The common weaknesses found across studies were the validity and reliability of the measurement of the exposures and outcomes. The weaknesses of the exposure were the use of interviews rather than objective assessments of chewing ability^35^^,^^38^ (eg, by color-changeable chewing gum test^37^ or dental status by clinical oral examinations).^15^ The details of method validation in some studies were also not stated clearly enough to determine study quality.^36^^,^^37^ The self-reported outcome of food intake increased the risk of bias from the possibility of under- or overreporting, which can affect the accuracy of the calculated daily nutrient intakes.^22^^,^^40^ Five studies clearly reported an attempt to minimize this effect by either using trained nutritionists^36^^,^^37^ or trained interviewers^15^^,^^30^^,^^38^ to interview participants about dietary information.

### Narrative synthesis: the associations of edentulism and dental prosthesis with nutrient intake

Dental prostheses are artificial appliances used to replace and restore damaged and dysfunctional teeth. Dentures are commonly used dental prostheses that can be categorized into 2 main types: complete dentures, which are used to restore complete edentulism; and partial dentures, which are used for partial edentulism.^41^ Prosthetic teeth provide an alternative for partial edentulism and involve surgically implanting individual artificial teeth into the jawbone to replace missing teeth on a more permanent basis.^41^^,^^42^ In a study, participants with prosthetic teeth were less likely to meet the daily requirements of macronutrients^36^ and had significantly lower daily energy intakes than older adults with natural sets of teeth.^32^ Although denture-wearing older adults can have difficulty chewing foods, having some natural teeth to support the dentures can result in their nutrient intakes not being significantly different from dentate older adults.^15^ Foods reported as “difficult to eat” included crusty bread, raw carrots, lettuce, apples, nuts, and well-cooked steak, with complete-denture wearers adding tomatoes and oranges to the list.^15^

In the study by Han and Kim,^33^ edentulous older adults who did not wear dentures were at twice the risk of being undernourished than those who did. However, their protein consumption was still within the acceptable range.^33^ Milledge et al^36^ found that although older adults did not meet the energy requirements, their protein intake was appropriate for their age regardless of edentulism and prostheses status. The carbohydrate and fat intake ratio was reported to be outside the acceptable range in both studies.^33^^,^^36^ Older adults with fewer teeth were at risk of not ingesting enough dietary fiber^15^; as the numbers of those with inadequate daily intakes were higher than for dentate participants.^36^ This corresponds to the findings that edentulous denture wearers consume fewer vegetables,^15^^,^^38^ fats,^32^ and oily fish.^15^

Edentate denture wearers had significantly lower intakes of calcium and thiamine than dentate nondenture wearers.^32^^,^^33^ Potassium, vitamin A, thiamine, niacin, vitamin C, and riboflavin were additionally reported by Han and Kim^33^ as being affected by edentulism and denture status. In the case of calcium and riboflavin, Han and Kim^33^ discussed that edentulism might not be associated, because deficiency was already observed within the sample population. The daily intake of vitamin C and potassium was likewise reported by Watson et al^15^ as significantly less consumed by edentate older adults with dentures than dentate older adults. Milledge et al^36^ did not report a significant difference between the micronutrient intakes of older adults based on edentulism and prostheses.

### Narrative synthesis: the associations of chewing ability with nutrient intake

Chewing difficulties were evaluated either by a self-administered questionnaire^35^^,^^38^ or by using color-changing chewing gum, whereby the more the color changes, the better the chewing ability.^37^ Chewing difficulty was associated with age, with complications especially reported by adults aged 75 years or older.^35^^,^^37^ Older adults with poor chewing ability had a significantly lower average number of functional teeth and blood serum albumin level (an indicator of undernutrition) than older adults with good chewing ability.^37^ Motokawa et al^37^ reported that the change in color of the chewing gum was associated with blood serum albumin level, but the number of teeth was not. This suggests that chewing ability, as determined by color-changing chewing gum, can better predict undernutrition than does the number of teeth.^37^

Those who reported chewing difficulties consumed significantly fewer fruits and vegetables^35^^,^^37^^,^^38^ but higher volumes of liquid beverages than those without.^35^ The amount of meat consumed was lower than eaten by the average adult but was not significantly different between older adults with different chewing abilities.^35^ Kwon et al^35^ reported that chewing difficulties significantly reduced the daily intake of vitamin C and potassium from what was already under the recommended values in older adults without chewing difficulty. Vegetables, fruits, and meat are the source of these nutrients. Motokawa et al^37^ additionally reported less intake of potatoes, seaweed, nuts, beans, seafood, and meat, but not of the cereal and sugar food groups. This corresponds with what was observed in edentulous older adults as previously discussed in the previous section of this review. The calculated daily nutrient intakes further reflect this finding, because carbohydrate was the only nutrient not significantly different among older adults with or without chewing difficulties. In contrast, the daily intake of all other nutrients investigated was lower in those with difficulty chewing than those without and was especially significant for protein, fats, iron, vitamin A, and vitamin C intake.^37^

### Narrative synthesis: the associations of occlusal force with nutrient intake

Inomata et al^34^ explored the effects of occlusal force on nutrient intake by older adults. Older men had higher levels of occlusal force than older women. Those with lower occlusal force tended to live with their family rather than alone or with their spouse. The occlusal force was not associated with body mass index of or energy intake by older adults. Individuals with higher levels of occlusal force ate significantly more vegetables, fish, and shellfish.^34^ Moreover, protein, polyunsaturated fatty acid, and dietary fiber intakes increased with occlusal force but not intake of fat and carbohydrates.^34^ Additionally, the daily intake of micronutrients, especially magnesium, phosphorus, iron, and zinc, increased significantly (*P *<* *.001) as the occlusal force increased.^34^

### Narrative synthesis: the associations of the number of teeth with nutrient intake

Counting one’s number of teeth is a simple method widely used to assess oral conditions.^34^ However, Inomata et al^34^ reported finding no associations between body mass index, energy intake, or food consumed and the number of teeth in older adults. Only polyunsaturated fatty acid was reported to be significantly associated with the number of teeth.^34^ Kim and Jin^30^ explored the effects of living status and the number of permanent teeth on the nutrient intake of older adults. Overall, older adults who lived alone had lower nutrient intakes than those with the same number of permanent teeth who lived with at least 1 other person. The lower protein and fat intake may be worsened if these individuals also have fewer teeth.^30^ Findings from both studies suggest macronutrient intakes may be affected more by living arrangements, such as living alone or with others, than by the number of teeth.

For micronutrients, Kim and Jin^30^ reported the intake of calcium and phosphorus was significantly lower among older adults living alone who had fewer than 11 teeth than among those with 21 or more teeth. Older adults with 11 to 20 teeth had significantly less intake of riboflavin and vitamin A than those with more teeth.^30^ However, this was not consistently seen in older adults with fewer than 11 teeth, so it may not have been caused by the number of teeth. Han and Kim^33^ further explained that riboflavin deficiency was a characteristic already observed within the sample population.

### Meta-analysis: the associations of denture with nutrient intake

Our meta-analysis of denture status subgroup and total energy intake included 3 studies with 3494 participants (control group, *n* = 1464, comparison group, *n* = 2030); that of protein, fat, carbohydrate, calcium, iron, vitamin C, and potassium intakes included 2 studies with 1714 participants (control group, *n* = 824, comparison group, *n* = 890).

**Figure 2.**
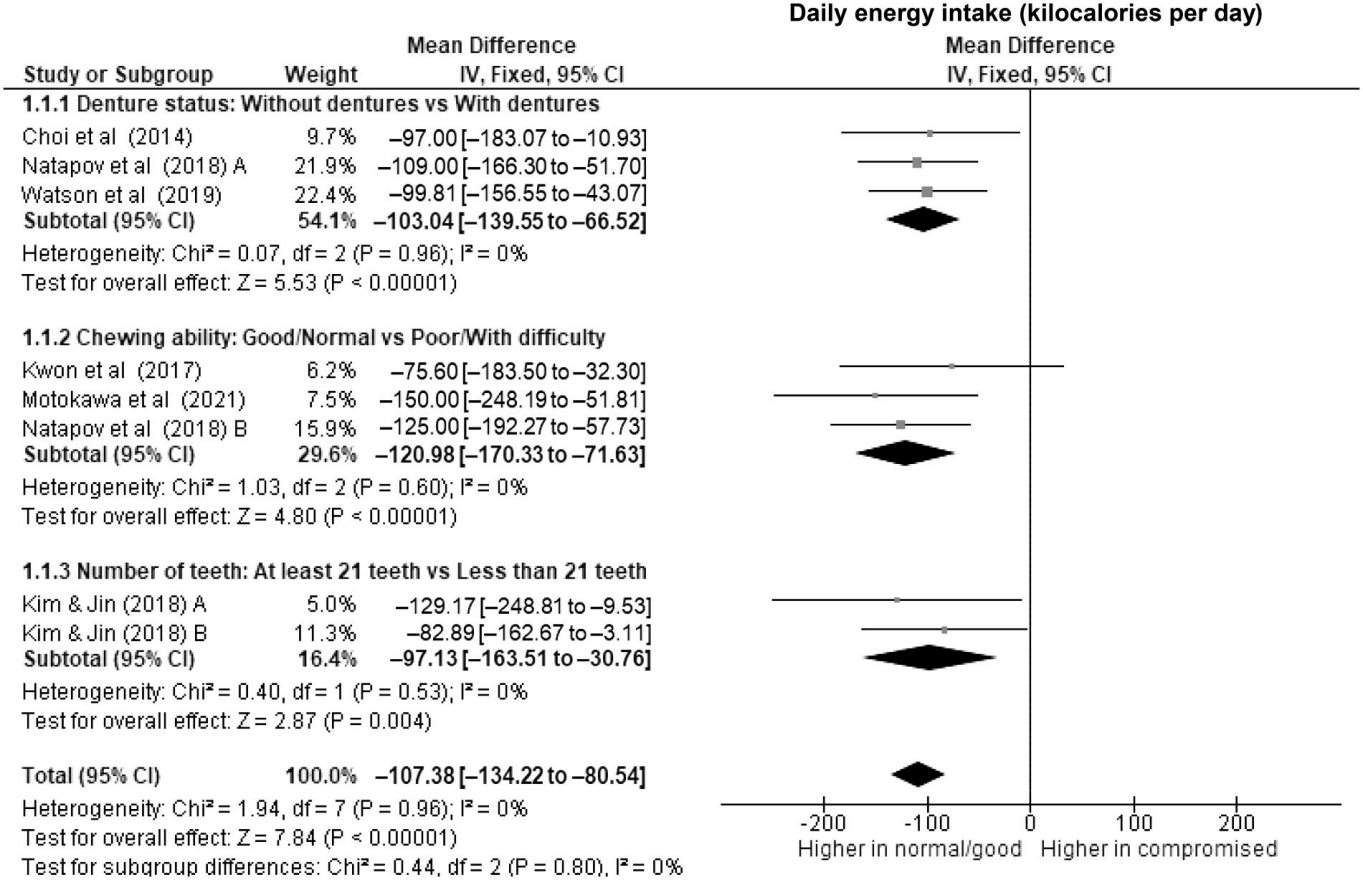
Forest Plot Showing the Overall and Subgroup Analysis for the Effects of Compromised oral Factors on Daily Energy Intake (kcal d^–1^) in Older Adults. *Abbreviations:* df, degrees of freedom; IV, inverse variance.

**Figure 3.**
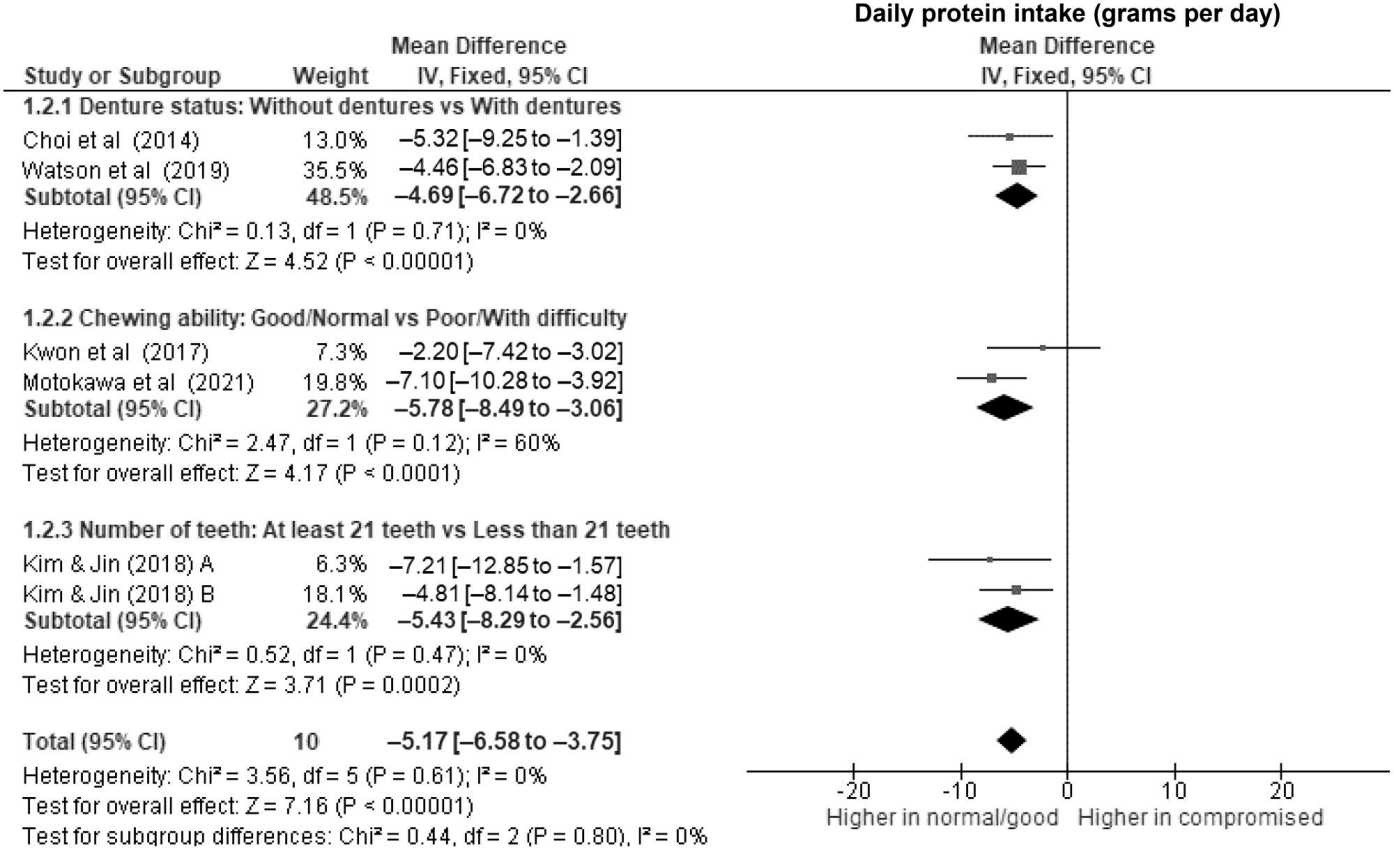
Forest Plot Showing the Overall and Subgroup Analysis for the Effects of Compromised Oral Factors on Daily Protein Intake (g d^–1^) in Older Adults. *Abbreviations:* df, degrees of freedom; IV, inverse variance.

**Figure 4.**
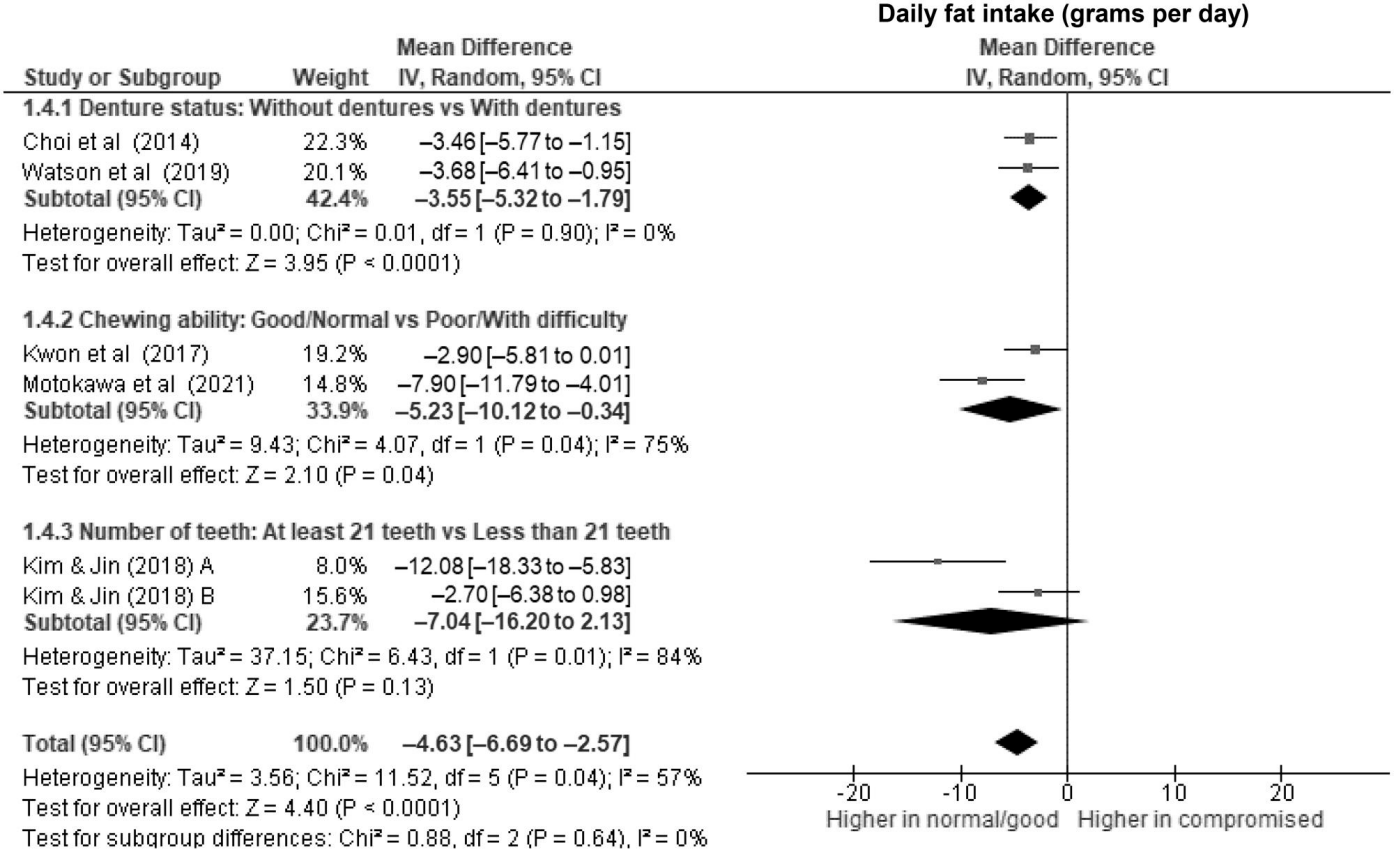
Forest Plot Showing the Overall and Subgroup Analysis for the Effects of Compromised Oral Factors on Daily Fat Intake (g d^–1^) in Older Adults. *Abbreviations:* df, degrees of freedom; IV, inverse variance.

**Figure 5.**
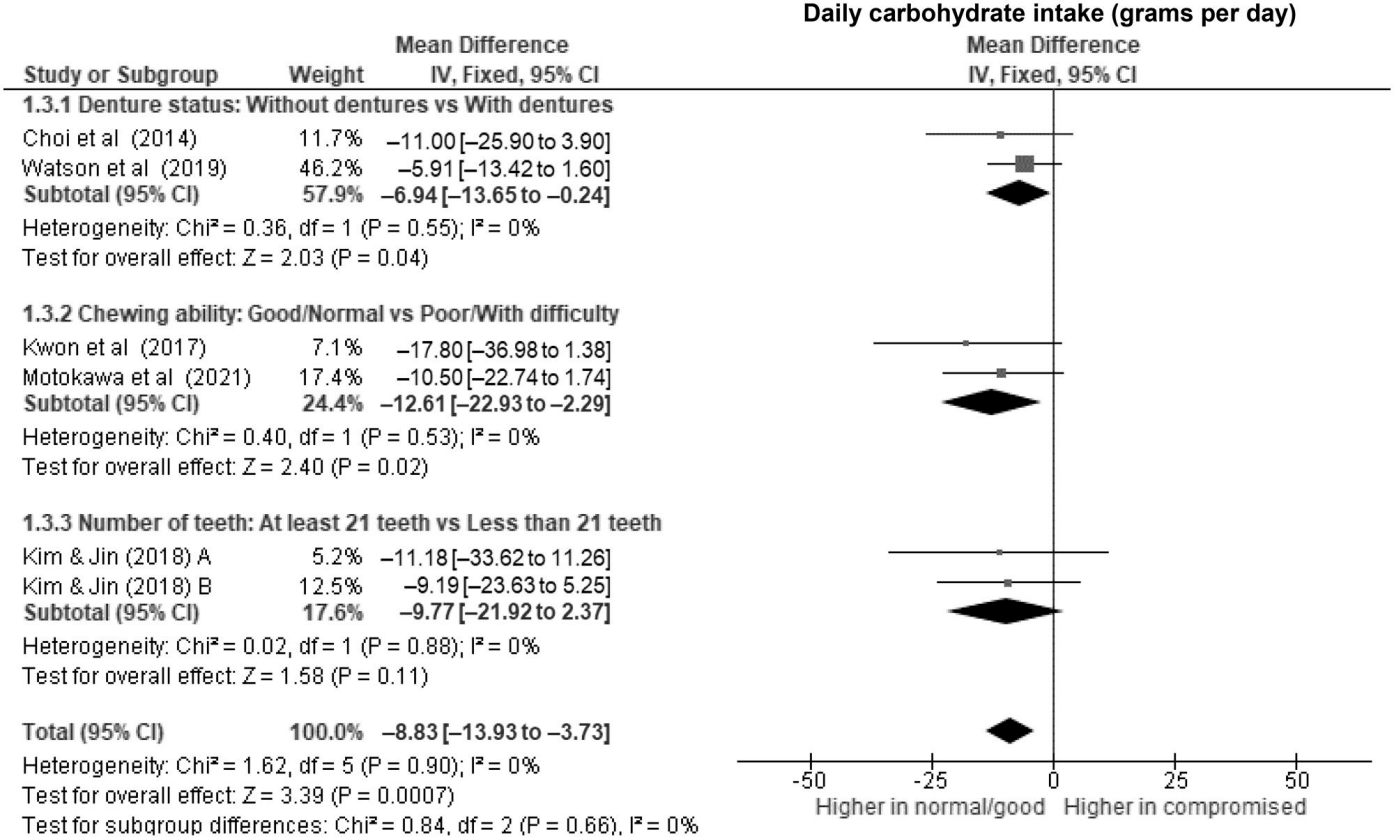
Forest Plot Showing the Overall and Subgroup Analysis for the Effects of Compromised Oral Factors on Daily Carbohydrate Intake (g d^–1^) in Older Adults. *Abbreviations:* df, degrees of freedom; IV, inverse variance.

**Figure 6.**
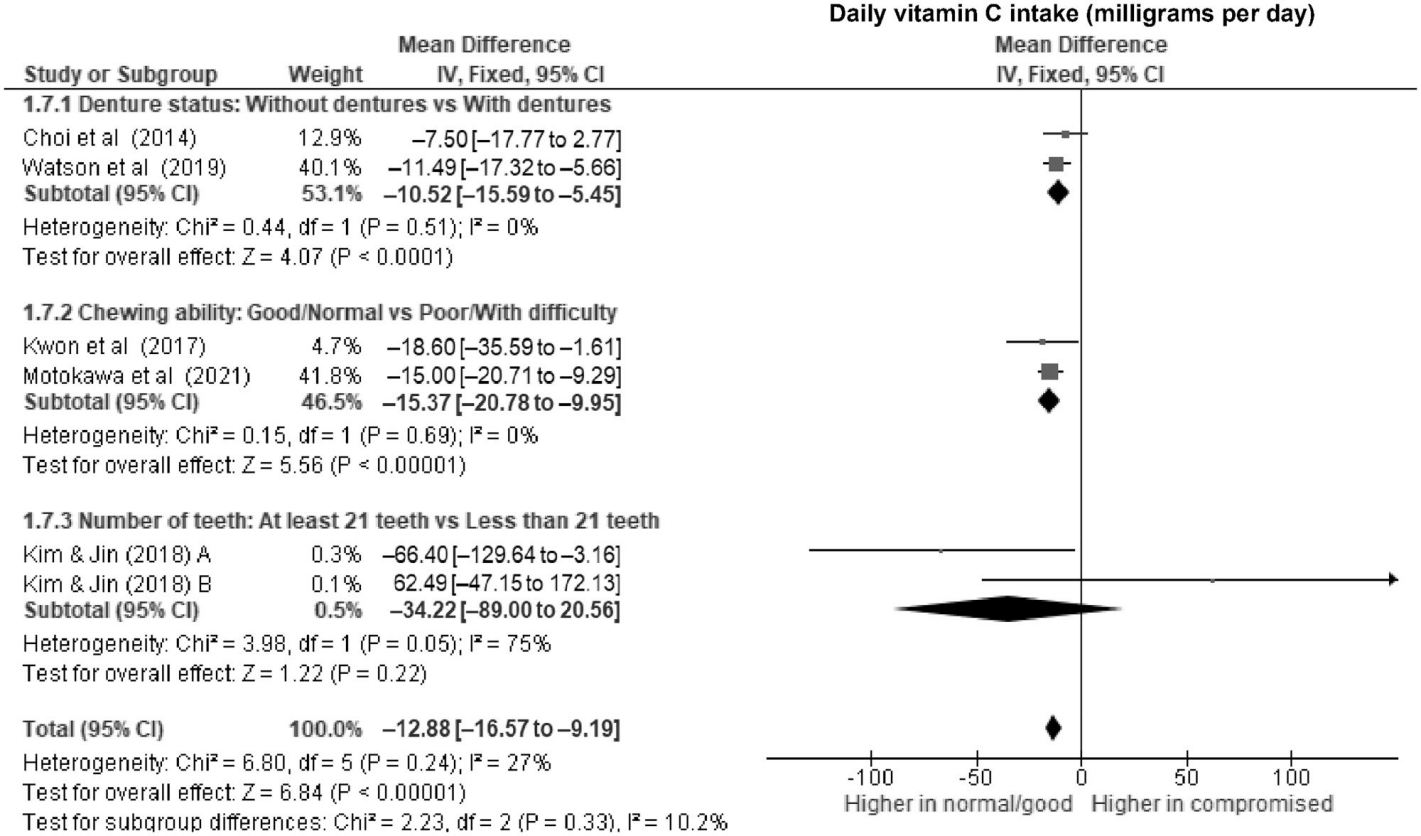
Forest Plot Showing the Overall and Subgroup Analysis for the Effects of Compromised Oral Factors on Daily Vitamin C Intake (mg d^–1^) in Older Adults. *Abbreviations:* df, degrees of freedom; IV, inverse variance.

Older adults with dentures had significantly lower daily energy intake (Figure 2), at 103 kcal d^–1^ (WMD, –103 kcal d^–1^; 95% CI, –140 to –67; *P *<* *.00001; *I*^2^ = 0%), along with lower protein intake (Figure 3) of nearly 5 g d^–1^ (WMD, –4.7 g d^–1^; 95% CI, –6.7 to –2.7; *P *<* *.00001; *I*^2^ = 0%) compared with those without dentures. Wearing dentures was also significantly associated with lower daily intake of fat (WMD, –3.6 g d^–1^; 95% CI, –5.3 to –1.8; *P *<* *.0001; *I*^2^ = 0%) (Figure 4), vitamin C (WMD, –10.5 mg d^–1^; 95% CI, –15.6 to –5.5; *P *<* *.0001; *I*^2^ = 0%) (Figure 6), dietary iron (WMD, –0.8 mg d^–1^; 95% CI, –1.2 to –0.5; *P *<* *.0001; *I*^2^ = 0%) (Figure S1), and potassium (WMD, –122.7 mg d^–1^; 95% CI, –209.6 to –35.8; *P *=* *.006; *I*^2^ = 0%) (Figure S2), although to a lesser extent. Evidence of associations between using a dental prosthesis and daily intake of carbohydrate (Figure 5) and calcium (Figure S3) was insufficient (*P *>* *.01).

### Meta-analysis: the associations of chewing ability with nutrient intake

The meta-analysis of the chewing ability subgroup and total energy intake included 3 studies with 3288 participants (control group, *n* = 2126, comparison group, *n* = 1162); that of protein, fat, carbohydrate, calcium, iron, and vitamin C intakes included 2 studies with 1508 participants (control group, *n* = 730, comparison group, *n* = 778).

Of all subgroups, older adults with and without compromised chewing ability had the largest difference in the daily energy intake (Figure 2), with a WMD of –121 kcal d^–1^ (95% CI, –170 to –72; *P *<* *.00001; *I*^2^ = 0%) and significantly lower protein intake of nearly 6 g d^–1^ (WMD, –5.8 g d^–1^; 95% CI, –8.5 to –3.1; *P *<* *.0001; *I*^2^ = 60%) (Figure 3) in those with poor chewing ability. For micronutrients, lower daily intakes of vitamin C (WMD, –15.4; 95% CI, –20.8 to –10.0; *P *<* *.00001) (Figure 6) and calcium (WMD, –58.5; 95% CI, –86.6 to –30.4; *P *<* *.0001) (Figure S3) were significantly associated with compromised chewing ability in older adults.

### Meta-analysis: the associations of the number of teeth with nutrient intake

The meta-analysis of the number-of-teeth subgroup with the total energy, protein, fat, carbohydrate, calcium, iron, vitamin C, and potassium intakes included 4 studies with 2904 participants (control group, *n* = 1571, comparison group, *n* = 1333).

Older adults with fewer than 21 teeth had lower daily energy intake (Figure 2) of 97 kcal d^–1^ (WMD, –97 kcal d^–1^; 95% CI, –164 to –31; *P* = .004; *I*^2^ = 0%) and lower daily protein intake of 5 g d^–1^ (WMD, –5.4; 95% CI, –8.3 to –2.6; *P* = .0002; *I*^2^ = 0%) (Figure 3) than those with more teeth. However, evidence of associations with daily fat (WMD, –7.0; 95% CI, –16.2 to –2.1; *P* = .13; *I*^2^ = 84%) (Figure 4), carbohydrates (WMD, –9.8; 95% CI, –21.9 to 2.4; *P* = .11; *I*^2^ = 0%) (Figure 5), and micronutrient intake were insufficient (*P *>* *.01).

### Meta-analysis: the associations of oral factors with nutrient intake

Previous studies have suggested positive relationships between the number of natural teeth, denture status, and masticatory function. Participants with more natural teeth had better chewing ability, which was further influenced by the type of denture used to replace missing teeth.^43^^,^^44^ Having fewer natural teeth extended beyond affecting chewing ability, further influencing oral health-related quality of life, a subjective assessment of how an individual's oral health influences their overall well-being and daily activities.^45^ Denture status also played a substantial role in these correlations.^46^ To explore the influence of these factors, the meta-analysis was conducted with all oral factors combined.

Combining the effect size of all oral factors by nutrient resulted in the inclusion of 8 studies (details in Table S6) with a total of 9686 participants (control group, *n* = 5161, comparison group, *n* = 4525) for data on the daily energy intake. The daily protein, fat, carbohydrate, calcium, iron, and vitamin C intakes included 6 studies with 6126 participants (control group, *n* = 3125, comparison group, *n* = 3001), and potassium intake included 4 studies with a sample size of 4618 participants (control group, *n* = 2395, comparison group, *n* = 2223).

Older adults with compromised oral factors have significantly lower daily energy (Figure 2) and protein intake (Figure 3), with differences of 107 kcal d^–1^ (WMD, –107 kcal d^–1^; 95% CI, –134 to –81; *P *<* *.00001; *I*^2^ = 0%) and 5 g d^–1^ (WMD, –5.2 g d^–1^; 95% CI, –6.6 to –3.8; *P *<* *.00001; *I*^2^ = 0%), respectively, compared with those without compromised oral factors. The daily fat (Figure 4) and carbohydrate (Figure 5) intakes were significantly lower in those with compromised oral factors. However, the differences were less pronounced, with smaller effect sizes of –4.6 g d^–1^ (95% CI, –6.7 to –2.6; *P *<* *.0001; *I*^2^ = 57%) for fat and –8.8 g d^–1^ (95% CI, –13.9 to –3.7; *P *=* *.0007; *I*^2^ = 0%) for carbohydrate intakes, respectively.

From the meta-analysis of micronutrients, lower daily intake of vitamin C (WMD, –12.9 mg d^–1^; 95% CI, –16.6 to –9.2; *P *<* *.00001; *I*^2^ = 27%) (Figure 6) was significantly associated with compromised oral factors in older adults. However, the daily intakes of dietary iron (WMD, –1.5 mg d^–1^; 95% CI, –2.8 to –0.3; *P *=* *.01; *I*^2^ = 88%) (Figure S1), calcium (WMD, –29.7 mg d^–1^; 95% CI, –64.9 to 5.5; *P *=* *.10; *I*^2^ = 70%) (Figure S3), and potassium (WMD, –92.5 mg d^–1^; 95% CI, –169.0 to –15.9; *P *=* *.02; *I*^2^ = 42%) (Figure S2) did not provide sufficient evidence to infer associations, because their *P* values were high.

From the sensitivity analysis, the exclusion of the studies with participants known to live with at least 1 other family member increased the strength of the association of oral factors and lower daily micronutrient intakes (Figures S4–S7). This was especially seen with the daily calcium and potassium intake. There was a decrease in overall WMD of daily calcium intake from –29.7 mg d^–1^ (95% CI, –64.9 to 5.5; *P *=* *.10; *I*^2^ = 70%) to –46.1 mg d^–1^ (95% CI, –65.2, –27.0; *P *<* *.00001; *I*^2^ = 0%). The overall WMD of the potassium intake decreased from –92.5 mg d^–1^ (95% CI, –169.0 to –15.9; *P *<* *.02; *I*^2^ = 42%) to –129.5 mg d^–1^ (95% CI, –213.3 to –45.8; *P *=* *.002; *I*^2^ = 0%) after the exclusion.

## DISCUSSION

This systematic review consists of 9 research articles, 6 of which were included in the meta-analysis of the daily energy intake, pooling a total number of 9686 healthy, community-dwelling, older adult participants. The meta-analyses of daily protein, fat, carbohydrate, calcium, iron, and vitamin C intakes included 5 articles, with 6126 healthy older adult participants per analysis, and the meta-analysis of potassium intake included 3 articles with 4618 older adult participants.

A significant association was found between compromised oral factors and lower daily intakes of energy and macronutrients by older adults. From all the micronutrients analyzed, only the daily intake of vitamin C was significantly associated with compromised oral factors. Meta-analyses of micronutrients also showed higher levels of heterogeneity than that of the energy and macronutrient intakes. Denture status alone was significantly associated with lower daily energy and intakes of all the macronutrients analyzed, but not of the micronutrients. When considering the variability of the WMD, denture status produced fewer variations with narrow CIs for effect estimates. This suggests there was less internal variability, because denture status can be easily and straightforwardly determined by a trained professional or other person. Heterogeneity observed between studies within a subgroup may be influenced by pooling studies that accounted for edentulism^15^ with those that did not.^32^^,^^38^ For older adults with poor chewing ability, apart from the daily energy and protein intakes, strong evidence of effects was also seen in the daily vitamin C and calcium intakes. The overall variability was more apparent in the study with self-assessed chewing ability^35^ than in the objectively assessed ability.^37^ Subjective and objective assessments of mastication are influenced by different factors,^47^ with subjective assessments exhibiting poorer prediction sensitivity and precision.^48^

Associations between a participant’s number of teeth and dietary intake were only significant for the total daily intakes of energy and protein. Compared with other methods in this meta-analysis, using the number of teeth to explore associations with food intake in older adults produced the highest variability. Although the number of teeth was examined by trained dentists, it may not accurately reflect actual eating capability, because the position of the missing tooth is also an important determinant of functionality.^49^ The exclusion of studies with participants living with family in the sensitivity analysis further increased the significance of associations between compromised oral conditions and lower daily micronutrient intakes, especially of calcium and potassium. This suggests that living status can influence the oral health and micronutrient intake of older adults. Previous studies have indicated that individuals living alone are more prone to experience poorer oral health,^30^^,^^50^ an increased risk of malnutrition,^51^ less dietary variety, and lower consumption of essential food groups.^52^^,^^53^

Poor oral conditions affect older adults’ perception of comfort when eating, which can lead to less food intake.^54^ Because the energy required to maintain bodily functions is gained through the consumption of protein, fat, and carbohydrate^55^, lower intakes will affect the total daily energy intake. Lower intake of vitamin C may have been affected by compromised oral processing factors: older adults with compromised oral processing factors often reported hard foods and those with fibrous structures such as fruits and vegetables, the source of vitamin C, as difficult to chew.^15^^,^^33^^,^^37^^,^^56–58^ Thus, older adults with compromised oral conditions may struggle to consume the required amounts of these nutrients. Chewing difficulties can be induced by the decline in masticatory efficiency in denture wearers and those with fewer teeth.^59^^,^^60^ A study showed that denture status alone did not have significant associations with nutrition status or eating enjoyment, unlike when it is considered together with edentulism.^33^^,^^61^ Edentulism affects nutrient intake, and prosthesis has been the main chosen treatment in clinical dentistry, with numerous studies conducted to support and improve its application.^62–65^ Although being dentate is the best condition for consuming nutrients, having more natural teeth and fixed prosthesis is better than having fewer natural teeth and removable dentures, according to study findings.^15^ Apart from poor fitting, the position of the removable denture or missing teeth can also affect mastication, because the contact area of the posterior teeth correlates with the bite force,^66^^,^^67^ and bite force significantly correlates with chewing efficiency regardless of the denture status.^68^ Similarly, chewing efficiency determined by the number of teeth alone is highly variable, and it is better to consider the type of teeth and functionality of the arrangement also. With more opposing tooth pairs, there will be more occlusal contacts and, thus, better masticatory potential.^49^^,^^69^ Inomata et al^34^ observed more significant differences in dietary nutrient intakes when older adults were grouped by their occlusal force rather than when grouped by the number of teeth.

The lack of literature on oral processing and its associations with nutrition intakes in older adults suggests that studies in this area were not extensive. However, it is worth exploring because findings from this review show that it can be a good representation of the eating ability in older adults. Studies tend to physically assess the oral cavity to study associations with nutrient intakes as a single element rather than in combination or by directly evaluating the performance. Furthermore, the Dietary Reference Intake values and the nutrition status of the general adult population within ae sampling country are critical to data interpretation because less intake is not necessarily related to equal to inadequate or deficient intake.^30^^,^^33^^,^^35^

### Strengths and limitations

The strength of this study lies in referencing the PRISMA guidelines and registering the review with PROSPERO for comprehensive and transparent reporting throughout the review, reducing the risk of bias that can occur from the selection, screening, and data extraction processes. The literature search was conducted extensively in a number of relevant databases, and the quality of the studies was rigorously assessed to identify all possible biases. The sample size of the meta-analyses was of sufficient power to observe effects with reasonable precision. The limitations of these meta-analyses come from the lack of population diversity. Although a large sample size was achieved even with a few studies, the settings mostly were in South Korea, and the results may not be generalized. The small number of eligible articles may limit the precision, because it affects the number of studies per subgroup, with the number of teeth subgroup ended up being composed only of studies extracted from the same research article. Nonetheless, they were different sets of individuals grouped according to their living status first and then by their number of teeth. This meta-analysis disregarded the individuals’ living status to increase the power to detect significant differences in nutrient intakes between studies based on the effects of the number of teeth alone. Variations in the exposure ascertainment, definition, and grouping criteria of each oral factor may explain the heterogeneity across the studies. Consumption of supplements was not accounted for in the studies and might be a possible source of residual confounding across studies and variability within the meta-analyses of micronutrients. Although the daily energy and nutrient intakes were significantly lower in older adults with compromised oral conditions, as explored, these findings do not imply a direct influence on the risk of malnutrition. This would require comparing the values with recommended daily intakes as appropriate to that population group to conclude whether this significant decrease affects the nutrition status, which was not the objective of this study, and interpretation of the results must be done with caution.

## CONCLUSIONS

In conclusion, there is significant evidence that denture status, chewing ability, and the number of natural teeth were associated with lower dietary intakes of energy, protein, fat, carbohydrate, and vitamin C, but the findings are based on a small number of studies. Due to the lack of studies covering all aspects of food oral processing (eg, salivary flow rate, tongue pressure), the associations between oral processing and nutrient intake could not be fully elucidated. Nonetheless, nutrition intake by older adults should be considered in association with their oral health, because a simple assessment of denture status may help identify those at risk of poor nutritional status.

## Supplementary Material

nuae080_Supplementary_Data
