# Supplementary material for: The Association of Oral Processing Factors and Nutrient Intake in Community-Dwelling Older Adults: A Systematic Review and Meta-Analysis
Source: Nutr Rev. 2024 Jun 25;83(3):e762–77. doi: 10.1093/nutrit/nuae080 (PMC11819486; doi:10.1093/nutrit/nuae080)
Supplement: nuae080_Supplementary_Data [file nuae080_supplementary_data.zip › nuae080_Supplementary_Data/8.pdf]

Supplementary material

Meta-analysis

The association of oral processing factors and nutrient intake in community-dwelling older adults: a systematic review and meta-analysis.

Supatchayaporn Nitsuwat[a], James Webster[b], Anwesha Sarkar[c], Janet Cade[d]

[a]https://orcid.org/0000-0002-6522-9813

[b]https://orcid.org/0000-0001-6999-6959

[c]https://orcid.org/0000-0003-1742-2122

[d]https://orcid.org/0000-0003-3421-0121

Affiliations: Supatchayaporn Nitsuwat, James Webster, and Janet Cade are with the Nutritional Epidemiology Group, School of Food Science and Nutrition, Faculty of Environment, University of Leeds, Leeds, UK. Anwesha Sarkar is with the Food Colloids and Bioprocessing Group, School of Food Science and Nutrition, Faculty of Environment, University of Leeds, Leeds, UK.

Corresponding author: Ms Supatchayaporn Nitsuwat, School of Food Science and Nutrition, Faculty of Environment, University of Leeds, Woodhouse Lane, Leeds LS2 9JT, United Kingdom. Email: fssn@leeds.ac.uk.

Supplementary material

Table S1. PRISMA checklist

**Section and Topic**	**Item #**	**Checklist item**	**Location where item is reported**
**TITLE**			
Title	1	Identify the report as a systematic review.	1
**ABSTRACT**			
Abstract	2	See the PRISMA 2020 for Abstracts checklist.	1
**INTRODUCTION**			
Rationale	3	Describe the rationale for the review in the context of existing knowledge.	3-4
Objectives	4	Provide an explicit statement of the objective(s) or question(s) the review addresses.	3-4
**METHODS**			
Eligibility criteria	5	Specify the inclusion and exclusion criteria for the review and how studies were grouped for the syntheses.	4
Information sources	6	Specify all databases, registers, websites, organisations, reference lists and other sources searched or consulted to identify studies. Specify the date when each source was last searched or consulted.	4
Search strategy	7	Present the full search strategies for all databases, registers and websites, including any filters and limits used.	4
Selection process	8	Specify the methods used to decide whether a study met the inclusion criteria of the review, including how many reviewers screened each record and each report retrieved, whether they worked independently, and if applicable, details of automation tools used in the process.	4
Data collection process	9	Specify the methods used to collect data from reports, including how many reviewers collected data from each report, whether they worked independently, any processes for obtaining or confirming data from study investigators, and if applicable, details of automation tools used in the process.	5
Data items	10a	List and define all outcomes for which data were sought. Specify whether all results that were compatible with each outcome domain in each study were sought (e.g. for all measures, time points, analyses), and if not, the methods used to decide which results to collect.	5

Supplementary material

Section and Topic	Item #	Checklist item	Location where item is reported
Data items (continue)	10b	List and define all other variables for which data were sought (e.g. participant and intervention characteristics, funding sources). Describe any assumptions made about any missing or unclear information.	5
Study risk of bias assessment	11	Specify the methods used to assess risk of bias in the included studies, including details of the tool(s) used, how many reviewers assessed each study and whether they worked independently, and if applicable, details of automation tools used in the process.	6
Effect measures	12	Specify for each outcome the effect measure(s) (e.g. risk ratio, mean difference) used in the synthesis or presentation of results.	6
Synthesis methods	13a	Describe the processes used to decide which studies were eligible for each synthesis (e.g. tabulating the study intervention characteristics and comparing against the planned groups for each synthesis (item #5)).	5-6
	13b	Describe any methods required to prepare the data for presentation or synthesis, such as handling of missing summary statistics, or data conversions.	5-6
	13c	Describe any methods used to tabulate or visually display results of individual studies and syntheses.	6
	13d	Describe any methods used to synthesize results and provide a rationale for the choice(s). If meta-analysis was performed, describe the model(s), method(s) to identify the presence and extent of statistical heterogeneity, and software package(s) used.	6
	13e	Describe any methods used to explore possible causes of heterogeneity among study results (e.g. subgroup analysis, meta-regression).	6
	13f	Describe any sensitivity analyses conducted to assess robustness of the synthesized results.	7
Reporting bias assessment	14	Describe any methods used to assess risk of bias due to missing results in a synthesis (arising from reporting biases).	5-6
Certainty assessment	15	Describe any methods used to assess certainty (or confidence) in the body of evidence for an outcome.	5-6

Supplementary material

Section and Topic	Item #	Checklist item	Location where item is reported
**RESULTS**			
Study selection	16a	Describe the results of the search and selection process, from the number of records identified in the search to the number of studies included in the review, ideally using a flow diagram.	7
	16b	Cite studies that might appear to meet the inclusion criteria, but which were excluded, and explain why they were excluded.	7
Study characteristics	17	Cite each included study and present its characteristics.	7
Risk of bias in studies	18	Present assessments of risk of bias for each included study.	8
Results of individual studies	19	For all outcomes, present, for each study: (a) summary statistics for each group (where appropriate) and (b) an effect estimate and its precision (e.g. confidence/credible interval), ideally using structured tables or plots.	8
Results of syntheses	20a	For each synthesis, briefly summarise the characteristics and risk of bias among contributing studies.	7-8
	20b	Present results of all statistical syntheses conducted. If metaanalysis was done, present for each the summary estimate and its precision (e.g. confidence/credible interval) and measures of statistical heterogeneity. If comparing groups, describe the direction of the effect.	12-16
	20c	Present results of all investigations of possible causes of heterogeneity among study results.	12-16
	20d	Present results of all sensitivity analyses conducted to assess the robustness of the synthesized results.	16
Reporting biases	21	Present assessments of risk of bias due to missing results (arising from reporting biases) for each synthesis assessed.	8
Certainty of evidence	22	Present assessments of certainty (or confidence) in the body of evidence for each outcome assessed.	8
**DISCUSSION**			
Discussion	23a	Provide a general interpretation of the results in the context of other evidence.	16-19
	23b	Discuss any limitations of the evidence included in the review.	19-20

Supplementary material

Section and Topic	Item #	Checklist item	Location where item is reported
Discussion (continue)	23c	Discuss any limitations of the review processes used.	19-20
	23d	Discuss implications of the results for practice, policy, and future research.	19-20
**OTHER INFORMATION**			
Registration and protocol	24a	Provide registration information for the review, including register name and registration number, or state that the review was not registered.	2, 4
	24b	Indicate where the review protocol can be accessed, or state that a protocol was not prepared.	4
	24c	Describe and explain any amendments to information provided at registration or in the protocol.	4
Support	25	Describe sources of financial or non-financial support for the review, and the role of the funders or sponsors in the review.	21
Competing interests	26	Declare any competing interests of review authors.	21
Availability of data, code and other materials	27	Report which of the following are publicly available and where they can be found: template data collection forms; data extracted from included studies; data used for all analyses; analytic code; any other materials used in the review.	4, 7

Supplementary material

Table S2. Search query in each included database.

**Web of Science:**<br>1. TI=("older adult*" or elderly or "aged care" or aging or ageing) or AB=("older adult*" or elderly or "aged care" or aging or ageing)<br>2. TI=("oral processing" or "chew*" or "mastication" or "bit* force" or "bit* strength" or "tongue pressure" or "tongue force" or dent* or saliva* or bolus or "saliva production" or "salivary flow") or AB=("oral processing" or "chew*" or "mastication" or "bit* force" or "bit* strength" or "tongue pressure" or "tongue force" or dent* or saliva* or bolus or "saliva production" or "salivary flow")<br>3. TI=("food intake" or "nutrient intake" or diet or dietary or "energy intake" or "food consumption") or AB=("food intake" or "nutrient intake" or diet or dietary or "energy intake" or "food consumption")<br>4. 1 and 2 and 3
**Scopus:** TITLE-ABS ("older adult*" OR elderly OR "aged care" OR aging OR ageing) AND TITLE-ABS ("oral processing" OR "bit* force" OR "bit* strength" OR "chew*" OR "mastication" OR "tongue force" OR "tongue pressure" OR dent* OR saliva* OR "salivary flow" OR bolus OR "saliva production") AND TITLE-ABS ("food intake" OR "nutrient intake" OR diet OR dietary OR "energy intake" OR "food consumption")
**Cochrane:** (older NEAR adult*) OR elderly OR "aged care" OR aging OR ageing in Title Abstract Keyword AND "oral processing" OR chew* OR mastication OR "bit* force" OR "bit* strength" OR "tongue pressure" OR "tongue force" OR dent* OR saliva* OR "salivary flow" OR "saliva production" OR bolus in Title Abstract Keyword AND "food intake" OR "nutrient intake" OR diet OR dietary OR "energy intake" OR "food consumption"
**Ovid (MedLine and Embase):**<br>1. ("older adult*" or elderly or "aged care" or aging or ageing).ti. or ("older adult*" or elderly or "aged care" or aging or ageing).ab.<br>2. ("oral processing" or "chew*" or "mastication" or "bit* force" or "bit* strength" or "tongue pressure" or "tongue force" or dent* or saliva* or bolus or "saliva production" or "salivary flow").ti. or ("oral processing" or "chew*" or "mastication" or "bit* force" or "bit* strength" or "tongue pressure" or "tongue force" or dent* or saliva* or bolus or "saliva production" or "salivary flow").ab.<br>3. ("food intake" or "nutrient intake" or diet or dietary or "energy intake" or "food consumption").ti. or ("food intake" or "nutrient intake" or diet or dietary or "energy intake" or "food consumption").ab.<br>4. 1 and 2 and 3
**CINAHL:**<br>S1: TI ("older adult*" or elderly or "aged care" or aging or ageing) OR AB ("older adult*" or elderly or "aged care" or aging or ageing)<br>S2: TI ("oral processing" or "chew*" or "mastication" or "bit* force" or "bit* strength" or "tongue pressure" or "tongue force" or dent* or saliva* or bolus or "saliva production" or "salivary flow") OR AB ("oral processing" or "chew*" or "mastication" or "bit* force" or "bit* strength" or "tongue pressure" or "tongue force" or dent* or saliva* or bolus or "saliva production" or "salivary flow")<br>S3: TI ("food intake" or "nutrient intake" or diet or dietary or "energy intake" or "food consumption") OR AB ("food intake" or "nutrient intake" or diet or dietary or "energy intake" or "food consumption")<br>S4: S1 AND S2 AND S3

Supplementary material

Table S3. Reports excluded with reason.

**Author** (Publication year)	**Title**
**Reason: Data of target group was combined with other groups** (n = 11)	
Bethene Ervin and Dye (2011)	Number of Natural and Prosthetic Teeth Impact Nutrient Intakes of Older Adults in the United States
Bousiou et al. (2021)	Oral factors and adherence to Mediterranean diet in an older Greek population
Cousson et al. (2012)	Nutritional status, dietary intake and oral quality of life in elderly complete denture wearers
Gaewkhiew et al. (2019)	Functional dentition, dietary intake and nutritional status in Thai older adults
Gaewkhiew et al. (2020)	Functional dentition and 12-month changes in dietary patterns among older adults in Thailand
Gupta et al. (2019)	Interrelationship between dental health status and nutritional status among elderly subjects in India
Habib et al. (2020)	Evaluation of Oral Hygiene and Dietary Status Among Edentulous, Dentate and Denture Wearers in the Elderly
Kagawa et al. (2012)	Effect of dental status and masticatory ability on decreased frequency of fruit and vegetable intake in elderly Japanese subjects
Mann et al. (2013)	The association between chewing and swallowing difficulties and nutritional status in older adults
Mosca et al. (2019)	How are macronutrient intake, BMI, ethnicity, age, and gender related to the composition of unstimulated saliva? A case study
Putri et al., (2020)	Masticatory Ability and Nutritional Status in Elderly Population
**Reason: Unhealthy population** (n = 7)	
Cin et al. (2021)	Evaluation of malnutrition status and related risk factors in geriatric outpatient clinic
El Hélou et al. (2014)	Relationship between oral health and nutritional status in the elderly: A pilot study in Lebanon
Iwasaki et al. (2016)	Hyposalivation and dietary nutrient intake among community based older Japanese
Kimura et al. (2013)	Evaluation of chewing ability and its relationship with activities of daily living, depression, cognitive status and food intake in the community-dwelling elderly
Pedersen et al., (2021)	Older age, smoking, tooth loss and denture-wearing but neither xerostomia nor salivary gland hypofunction are associated with low intakes of fruit and vegetables in older Danish adults
Su et al. (2020).	Denture Wearing and Malnutrition Risk Among Community Dwelling Older Adults
Takeuchi et al. (2014)	Nutritional status and dysphagia risk among Community dwelling frail older adults

Supplementary material

Table S3. Reports excluded with reason. (continue)

**Reason: Outcome not suitable** (n = 6)	
De Marchi et. al. (2012)	Association between number of teeth, edentulism and use of dentures with percentage body fat in south Brazilian community dwelling older people
Gaewkhiew et al. (2020)	Functional dentition and 12-month changes in body measurements among Thai older adults
Kotronia et al. (2021)	Poor oral health and the association with diet quality and intake in older people in two studies in the UK and USA
Lo et al. (2016)	Combined Effects of Chewing Ability and Dietary Diversity on Medical Service Use and Expenditures
Sato et al. (2016)	Ten-year longitudinal study on the state of dentition and subjective masticatory ability in community-dwelling elderly people
Aquilanti et al. (2020)	Impact of elderly masticatory performance on nutritional status: An observational study
**Reason: Institutionalized** (n = 5)	
Altenhoevel et al. (2012)	The impact of self-perceived masticatory function on nutrition and gastrointestinal complaints in the elderly
Banerjee et al. (2018)	Evaluation of relationship between nutritional status and oral health related quality of life in complete denture wearers
Petrovski et al. (2020)	Correlation of oral health and diet among institutionalized elderly people
Pohlhausen et al. (2016)	Energy and protein intake, anthropometrics, and disease burden in elderly home-care receivers - A cross-sectional study in Germany (ErnSIPP study)
Poisson et al. (2016)	Relationships between oral health, dysphagia and undernutrition in hospitalised elderly patients
**Reason: No control intervention** (n = 3)	
do Amaral et al. (2019)	Sensorial ability, mastication and nutrition of single-implant overdentures wearers
Gonçalves et al. (2015)	Effects of implant-based prostheses on mastication, nutritional intake, and oral health-related quality of life in partially edentulous patients: a paired clinical trial
Hamdan et al. (2013)	Do implant overdentures improve dietary intake? A randomized clinical trial
**Reason: Wrong intervention** (n = 2)	
Gupta et al. (2018)	Prevalence and risk factors of underweight, overweight and obesity among a geriatric population living in a high-altitude region of rural Uttarakhand, India
Suzuki et al. (2018)	The effect of new complete denture fabrication and simplified dietary advice on nutrient intake and masticatory function of edentulous elderly: A randomized-controlled trial
**Reason: Full-text not in English** (n = 1)	
Cusson et al. (2015)	Relation entre la mastication et la qualite de l'alimentation des aines de l'Etude longitudinale quebecoise sur la nutrition et le vieillissement (NuAge)

Supplementary material Table

Table S4. Metric of the results presented in the included studies that passed the screening criteria.

**Reference**	**Factors adjusted for**	**Reported results**			
		**Reporting format**	**Energy intake** (unit)	**Macronutrient intake** (unit)	**Micronutrient intake** (unit)
**Choi et al. (2014)**[1]	Sex and income.	Mean (arithmetic), and standard deviation (SD)	Energy (kcal/day)	Per day: Protein (g), fat (g), carbohydrate (g), and dietary fiber (g)	Per day: Ash (g), calcium (mg), phosphorus (mg), iron (mg), sodium (mg), potassium (mg), vitamin A (μg RE), carotene (μg), retinol (μg), thiamin (mg), riboflavin (mg), niacin (mg), and vitamin C (mg)
**Han and Kim (2016)**[2]	Age, sex, income, education, welfare receipt, and total energy intake.	Mean (arithmetic), and standard error (SE)	Energy (% Korean Dietary Reference Intakes, KDRI)	% KDRI: Protein (%)	%KDRI: Calcium (%), phosphorus (%), iron (%), sodium (%), potassium (%), vitamin A (%), thiamin (%), riboflavin (%), niacin (%), and Vitamin C (%)
**Inomata et al. (2017)**[3]	Sex, education, financial status, family structure, area of residence, and body mass index.	Median, and range	Energy (kcal/day)	% Energy intake: Protein (%), fat (%), mono-unsaturated fatty acid (%), polyunsaturated fatty acid (%), carbohydrates (%), cholesterol (mg), and dietary fiber (g)	Per 1000 kcal: Calcium (mg), phosphorus (mg), iron (mg), sodium (mg), potassium (mg), zinc (mg), vitamin A (μg RE), α-carotene (g), β-carotene (g), thiamin (mg), riboflavin (mg), niacin (mg), vitamin C (mg), vitamin D (μg), α-tocopherol (mg), vitamin K (μg), vitamin B6 (mg), vitamin B12 (mg), folate (μg), and pantothenic acid (mg)

Supplementary material

Table S4. Metric of the results presented in the included studies that passed the screening criteria. (continue)

**Reference**	**Factors adjusted for**	**Reported results**			
		**Reporting format**	**Energy intake** (unit)	**Macronutrient intake** (unit)	**Micronutrient intake** (unit)
**Kim and Jin (2018)**[4]	Age, sex, income, education, general health status, health behaviors, oral health status, and oral health behaviors.	Mean (geometric), and confidence interval (CI)	Energy (kcal/day)	Per day: Protein (g), fat (g), and carbohydrate (g)	Per day: Calcium (mg), phosphorus (mg), iron (mg), potassium (mg), vitamin A (µg RE), riboflavin (mg), and vitamin C (mg)
**Kwon et al. (2017)**[5]	Model 1: sex and age<br>Model 2: sex, age, and energy intake	Mean (arithmetic), and SE	Energy (kcal/day)	Per day: Protein (g), fat (g), saturated fatty acid (g), and carbohydrate (g)	Per day: Calcium (mg), phosphorus (mg), iron (mg), sodium (mg), potassium (mg), vitamin A (µg RE), thiamin (mg), riboflavin (mg), niacin (mg), and vitamin C (mg)
**Milledge et al. (2021)**[6]	None	Median, and 5th and 95th percentile	Energy (kJ/day)	Per day: Protein (as g, g per kg body weight, and % Energy intake), Carbohydrate (as g, and % Energy intake), Total fat (as g, and % Energy intake), Saturated fat (as g, and % energy intake), and dietary fiber (g)	Per day: Calcium (mg), phosphorus (mg), iron (mg), sodium (mg), potassium (mg), magnesium (mg), zinc (mg), iodine (µg), vitamin A (µg RE), thiamin (mg), riboflavin (mg), niacin equivalent (mg), vitamin C (mg), vitamin D (µg), vitamin E (mg), and dietary folate equivalent (µg)

Supplementary material

Table S4. Metric of the results presented in the included studies that passed the screening criteria. (continue)

**Reference**	**Factors adjusted for**	**Reported results**			
		**Reporting format**	**Energy intake** (unit)	**Macronutrient intake** (unit)	**Micronutrient intake** (unit)
**Motokawa et al. (2021)[7]**	None	Mean (arithmetic), and SD	Energy (kcal/day)	Per day: Protein (g), fat (g), and carbohydrate (g)	Per day: Calcium (g), iron (g), vitamin A (µg), thiamin (mg), riboflavin (mg), vitamin C (mg), and vitamin D (µg)
**Natapov et al. (2018)[8]**	Education, and the degree of interest in the relations of nutrition and health, and reading nutrition labels	Mean (arithmetic), and SD	Energy (kcal/day)	Per day: Protein (g), and fiber (g)	-
**Watson et al. (2019)[9]**	Age, sex, living status, socioeconomic status, energy intake, survey year, and smoking status	Mean (arithmetic), and SD	Energy (kJ/day)	Per day: Protein (g), fat (g), saturated fat (g), monounsaturated fat (g), omega-3 fatty acids (g), omega-6 fatty acids (g), carbohydrate (g), total sugars (g), and non-starch polysaccharides (g)	Per day: Calcium (mg), iron (mg), potassium (mg), magnesium (mg), iodine (µg), β-carotene (µg), retinol (µg), vitamin C (mg), vitamin D (µg), vitamin B6 (mg), vitamin B12 (µg), and folate (µg)

Supplementary material

Table S5. Risk of bias as assessed using Joanna Briggs Institute (JBI) Critical Appraisal tool checklist for analytical cross-sectional studies.

**Checklist items**	**Choi et al. (2014)[1]**	**Han and Kim (2016)[2]**	**Inomata et al. (2017)[3]**	**Kim and Jin (2018)[4]**	**Kwon et al. (2017)[5]**	**Milledge et al. (2021)[6]**	**Motokawa et al. (2021)[7]**	**Natapov et al. (2018)[8]**	**Watson et al. (2019)[9]**
**1. Were the criteria for inclusion in the sample clearly defined?**	No	Yes	Yes	No	Yes	Yes	No	Yes	Yes
**2. Were the study subjects and the setting described in detail?**	Yes	Yes	Yes	Yes	Yes	Yes	Yes	Yes	Yes
**3. Was the exposure measured in a valid and reliable way?**	Yes	Yes	Yes	Yes	No	Unclear	Unclear	No	No
**4. Were objective, standard criteria used for measurement of the condition?**	Yes	Yes	Yes	Yes	No	Yes	Yes	No	Yes
**5. Were confounding factors identified?**	Yes	Yes	Yes	Yes	Yes	Yes	No	Yes	Yes
**6. Were strategies to deal with confounding factors stated?**	Yes	Unclear	Yes	Yes	Yes	Yes	No	Yes	Yes
**7. Were the outcomes measured in a valid and reliable way?**	No	No	No	Yes	Unclear	Yes	Yes	No	Yes
**8. Was appropriate statistical analysis used?**	Yes	Yes	Yes	Yes	Yes	Yes	Yes	Yes	Yes
**Percentage of "yes" answers**	75% (High)	75% (High)	88% (High)	88% (High)	63% (Moderate)	88% (High)	50% (Moderate)	63% (Moderate)	88% (High)
**Overall appraisal**	**Include**	**Include**	**Include**	**Include**	**Include**	**Include**	**Include**	**Include**	**Include**
**Comments**	Concerns on dietary assessment method and poor reporting.	-	-	Concerns on dietary assessment method and poor reporting.	Concerns on dietary assessment method.	-	Concerns on dietary assessment method and poor reporting.	Concerns on dietary and denture status assessment.	Concerns on dietary and denture status assessment.

Supplementary material

Table S6 Summary of oral factor controls and comparison groups from the studies included in the meta-analysis by study ID.

Study ID	Control	Comparison
**Dentition status**		
Choi et al. (2014)[1]	No dentures	With dentures
Natapov et al. (2018) A[8]	No dentures	With dentures
Watson et al. (2019) A[9]	No dentures	With dentures, dentate<br>With dentures, edentate
**Chewing ability**		
Kwon et al. (2017)[5]	Normal	With difficulty
Motokawa et al. (2021)[7]	Good	Poor
Natapov et al. (2018) B[8]	Normal	With difficulty
**Number of teeth**		
Kim and Jin (2018) A[4]	≥ 21 teeth and lives alone	0-10 teeth and lives alone<br>11-20 teeth and lives alone
Kim and Jin (2018) B[4]	≥ 21 teeth and lives with family	0-10 teeth and lives with family<br>11-20 teeth and lives with family

Supplementary material

Reference

1. Choi YK, Park DY, Kim Y. Relationship between prosthodontic status and nutritional intake in the elderly in Korea: National Health and Nutrition Examination Survey (NHANES IV). *Int J Dent Hyg*. 2014;12(4):285-290. doi:10.1111/idh.12066

2. Han SY, Kim CS. Does denture-wearing status in edentulous South Korean elderly persons affect their nutritional intakes? *Gerodontology*. 2016;33(2):169-176. doi:10.1111/ger.12125

3. Inomata C, Ikebe K, Okubo H, et al. Dietary intake is associated with occlusal force rather than number of teeth in 80-y-old Japanese. *JDR Clin Trans Res*. 2017;2(2):187-197. doi:10.1177/2380084416673963

4. Kim EJ, Jin BH. Comparison of oral health status and daily nutrient intake between elders who live alone and elders who live with family: based on the Korean National Health and Nutrition Examination Survey (KNHANES VI) (2013-2015). *Gerodontology*. 2018;35(2):129-138. doi:10.1111/ger.12334

5. Kwon SH, Park HR, Lee YM, et al. Difference in food and nutrient intakes in Korean elderly people according to chewing difficulty: using data from the Korea National Health and Nutrition Examination Survey 2013 (6th). *Nutr Res Pract*. 2017;11(2):139-146. doi:10.4162/nrp.2017.11.2.139

6. Milledge K, Cumming RG, Wright FAC, et al. Associations between the composition of functional tooth units and nutrient intake in older men: the Concord Health and Ageing in Men Project. *Public Health Nutr*. 2021;24(18):6335-6345. doi:10.1017/S1368980021003566

7. Motokawa K, Mikami Y, Shirobe M, et al. Relationship between chewing ability and nutritional status in Japanese older adults: a cross-sectional study. *Int J Environ Res Public Health*. 2021;18(3). doi:10.3390/ijerph18031216

Supplementary material

8. Natapov L, Kushnir D, Goldsmith R, Dichtiar R, Zusman SP. Dental status, visits, and functional ability and dietary intake of elderly in Israel. *Isr J Health Policy Res*. 2018;7(1):58. doi:10.1186/s13584-018-0252-x
9. Watson S, McGowan L, McCrum L-A, et al. The impact of dental status on perceived ability to eat certain foods and nutrient intakes in older adults: cross-sectional analysis of the UK National Diet and Nutrition Survey 2008–2014. *Int J Behav Nutr Phys Act*. 2019;16(1):43. doi:10.1186/s12966-019-0803-8

Supplementary material

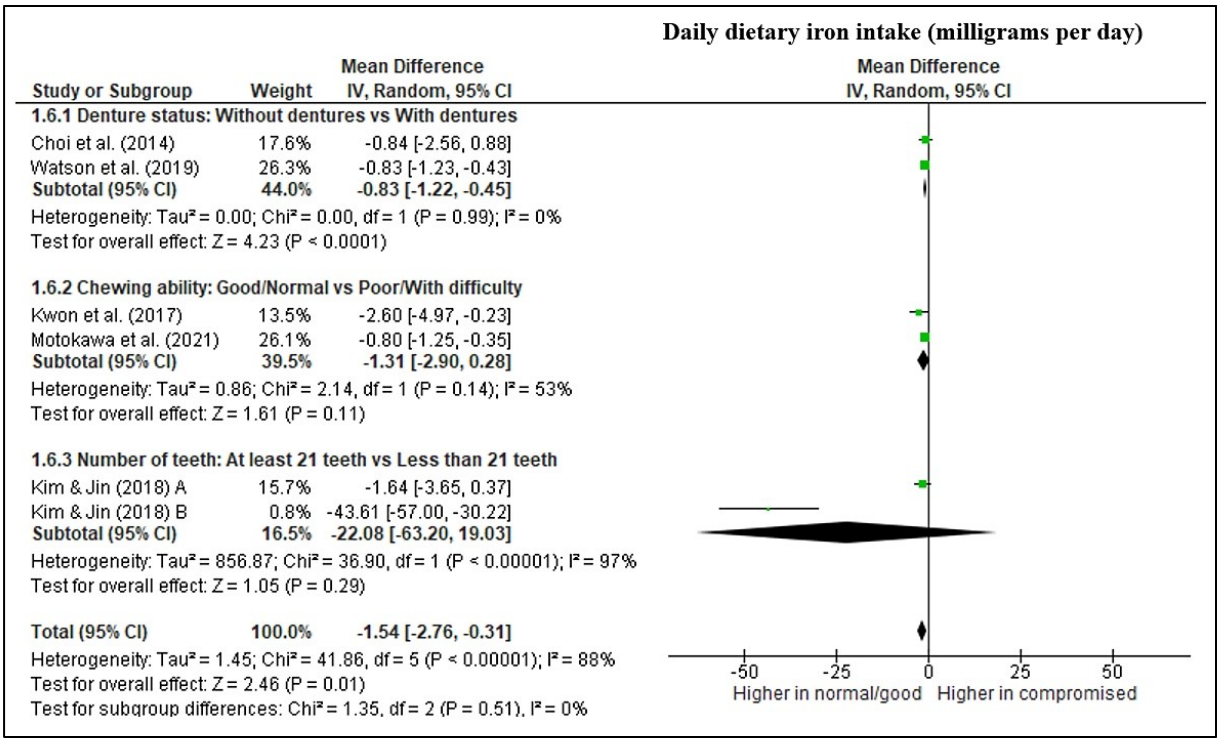

Figure S1. Forest plot comparing the difference in the daily dietary iron intake (mg/day) between older adults with normal or good oral processing and components to those with compromised oral processing and components.

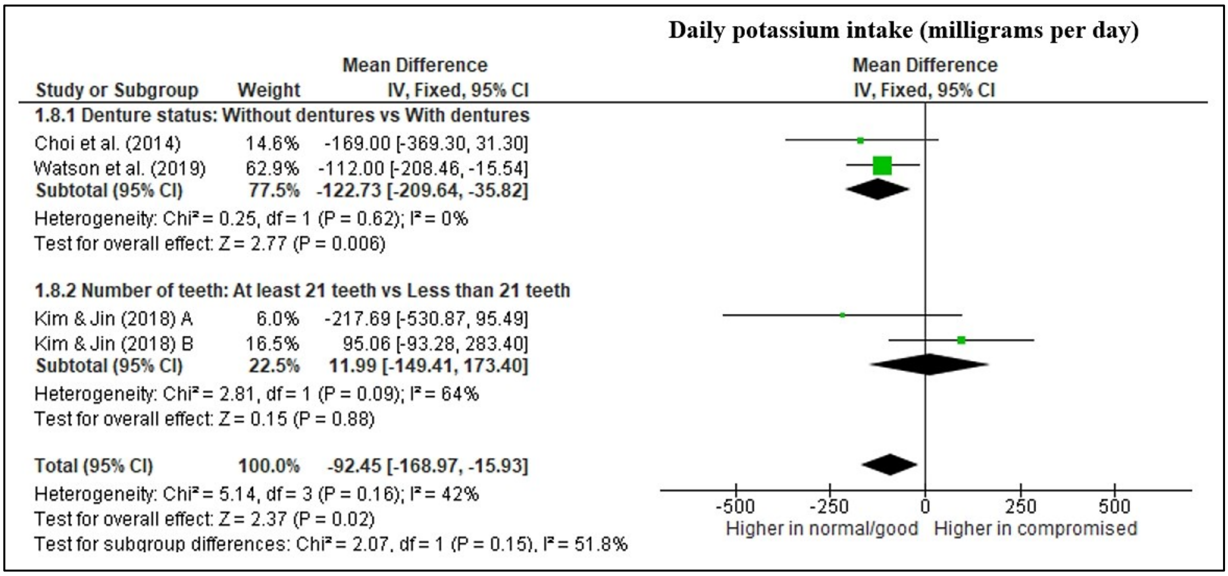

Figure S2. Forest plot comparing the difference in the daily potassium intake (mg/day) between older adults with normal or good oral processing and components to those with compromised oral processing and components.

Supplementary material

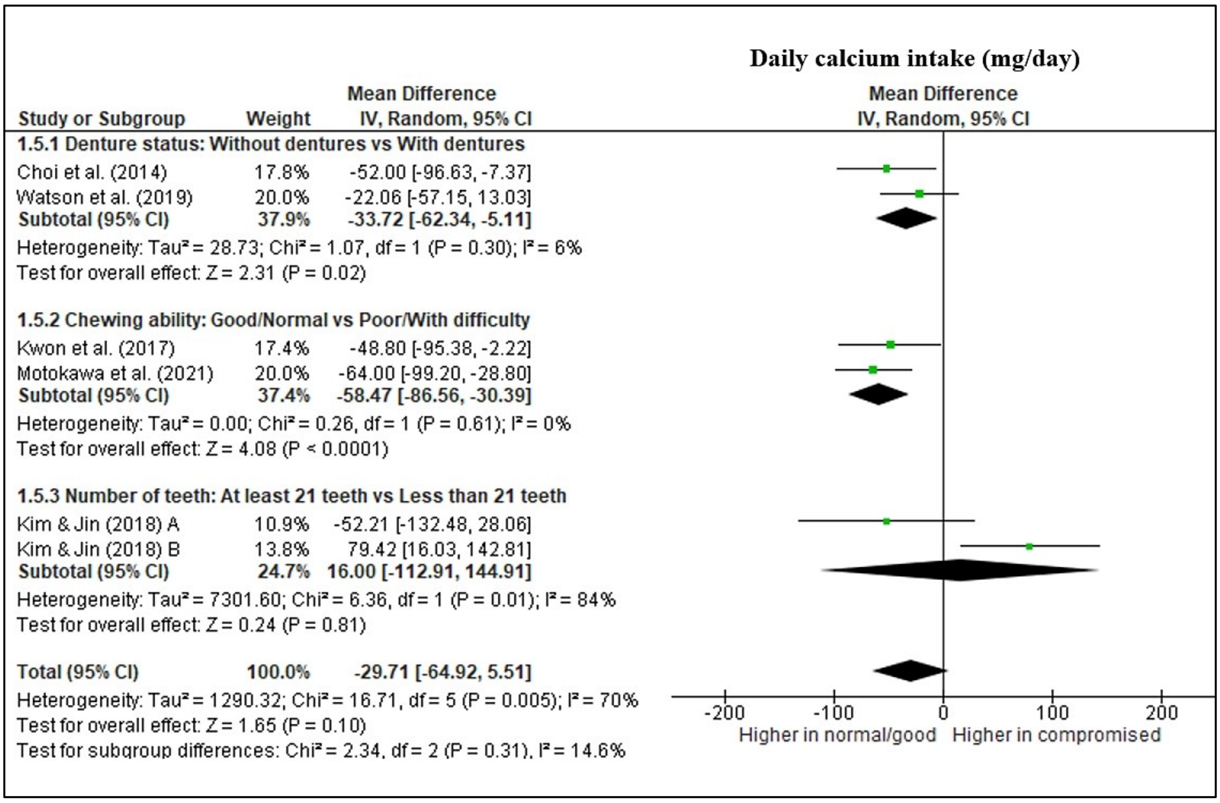

Figure S3. Forest plot comparing the difference in the daily calcium intake (mg/day) between older adults with normal or good oral processing and components to those with compromised oral processing and components.

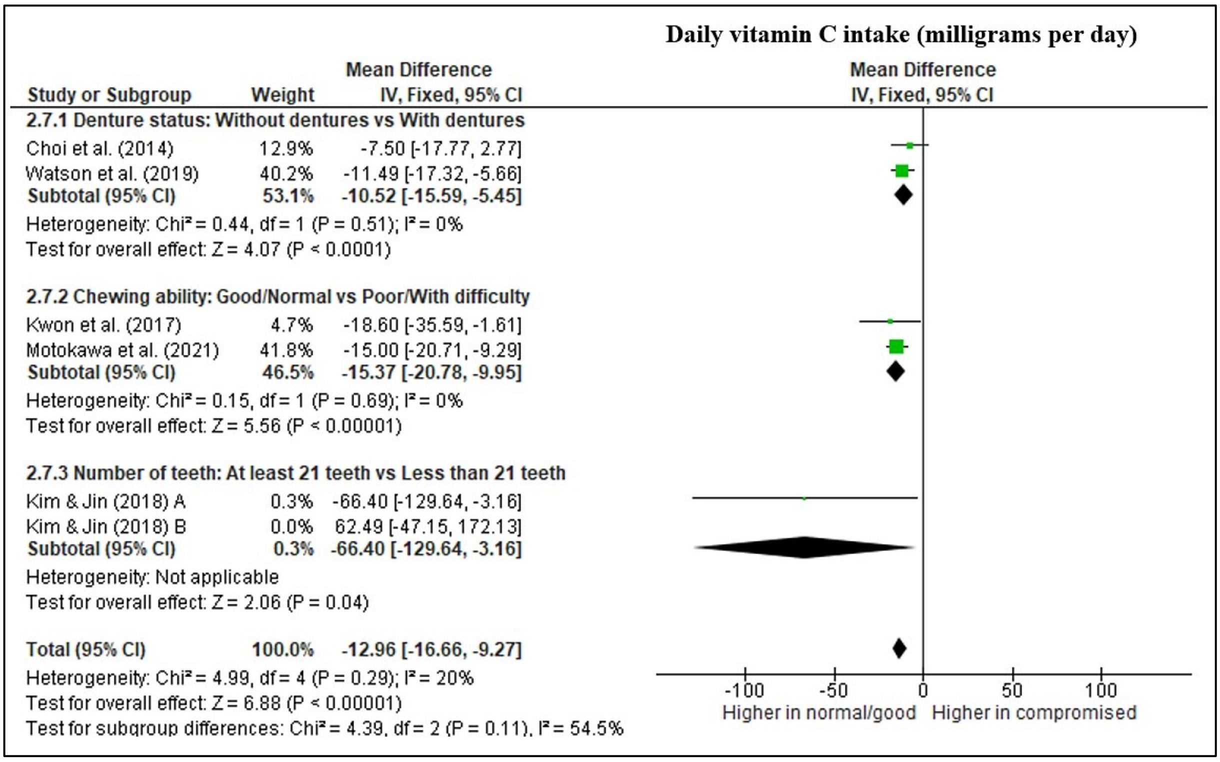

Figure S4. Forest plot from sensitivity analysis on the effects of compromised oral factors on daily vitamin C intake (mg/day) in older adults.

Supplementary material

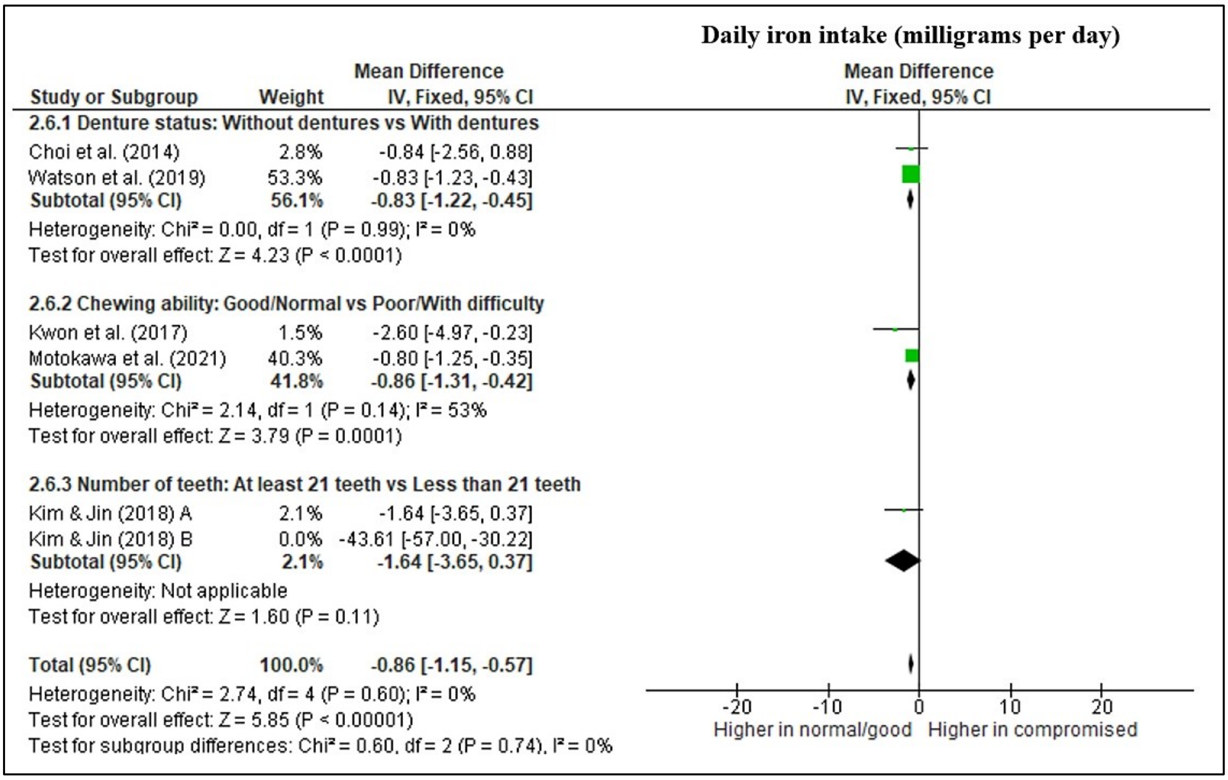

Figure S5. Forest plot from sensitivity analysis on the effects of compromised oral factors on daily dietary iron intake (mg/day) in older adults.

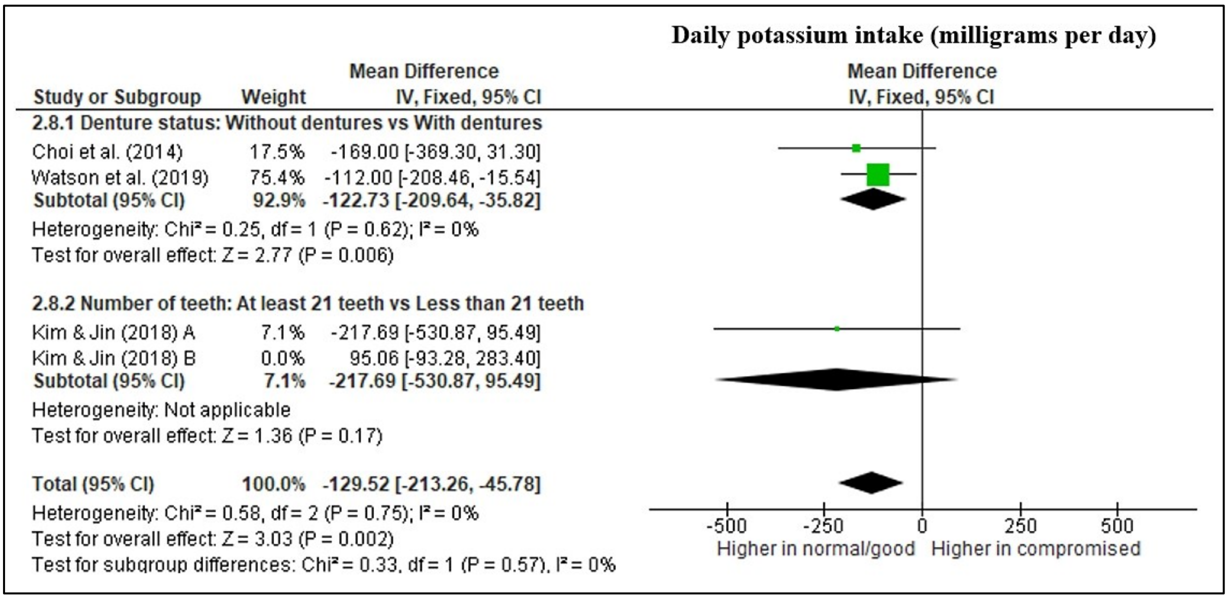

Figure S6. Forest plot from sensitivity analysis on the effects of compromised oral factors on daily potassium intake (mg/day) in older adults.

Supplementary material

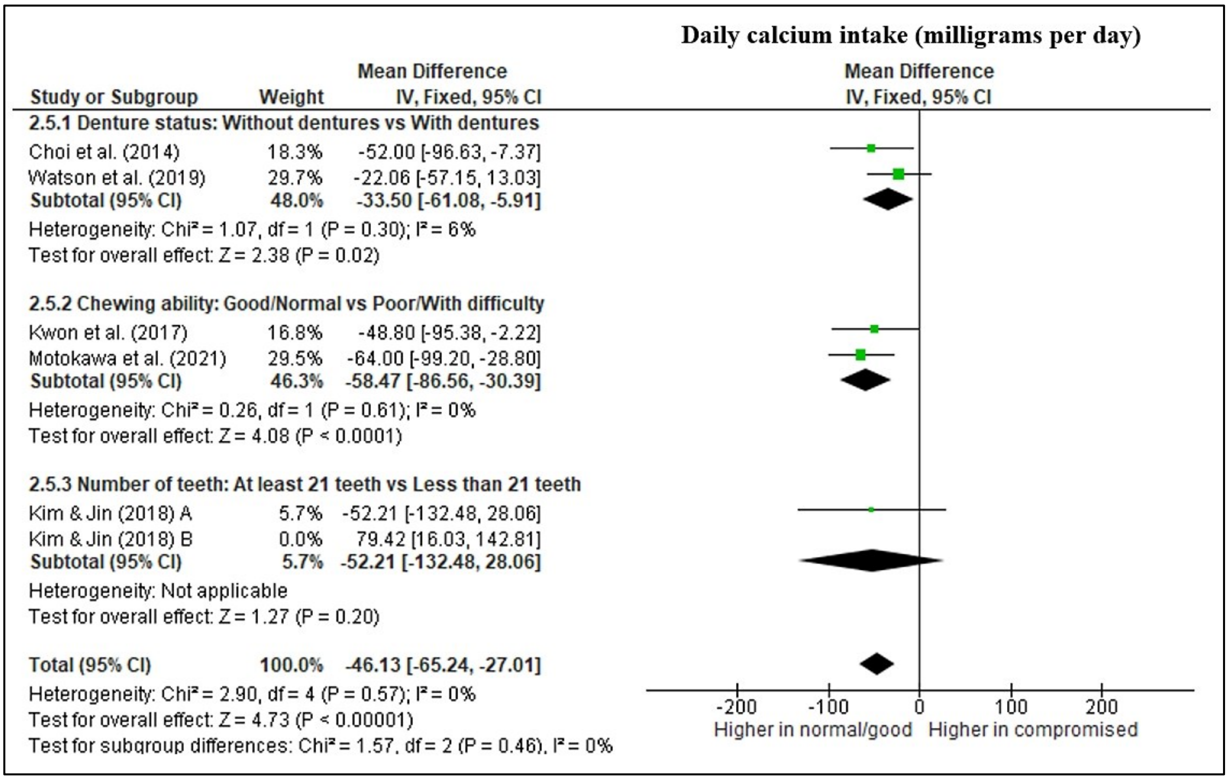

Figure S7. Forest plot from sensitivity analysis on the effects of compromised oral factors on daily dietary calcium intake (mg/day) in older adults.
